# Sociodemographic, mental health, and physical health factors associated with participation within re-contactable mental health cohorts: an investigation of the GLAD Study

**DOI:** 10.1186/s12888-023-04890-x

**Published:** 2023-07-26

**Authors:** Steven J. Bright, Christopher Hübel, Katherine S. Young, Shannon Bristow, Alicia J. Peel, Christopher Rayner, Jessica Mundy, Alish B. Palmos, Kirstin L. Purves, Gursharan Kalsi, Cherie Armour, Ian R. Jones, Matthew Hotopf, Andrew M. McIntosh, Daniel J. Smith, James T. R. Walters, Henry C. Rogers, Katherine N. Thompson, Brett N. Adey, Dina Monssen, Saakshi Kakar, Chelsea M. Malouf, Colette Hirsch, Kiran Glen, Emily J. Kelly, David Veale, Thalia C. Eley, Gerome Breen, Molly R. Davies

**Affiliations:** 1 Social, Genetic and Developmental Psychiatry Centre, Institute of Psychiatry, Psychology & Neuroscience, PO80, De Crespigny Park, Denmark Hill, London, SE5 8AF UK; 2 NIHR Maudsley Biomedical Research Centre, King’s College London, London, UK; 3 Department of Economics and Business Economics, National Centre for Register-Based Research, Aarhus University, Aarhus, Denmark; 4 Research Centre for Stress, Trauma & Related Conditions (STARC), School of Psychology, Queen’s University Belfast (QUB), Belfast, Northern Ireland UK; 5 Division of Psychiatry and Clinical Neurosciences, National Centre for Mental Health and MRC Centre for Neuropsychiatric Genetics and Genomics, Cardiff University, Cardiff, UK; 6 Institute of Psychiatry, Psychology and Neuroscience, King’s College London, London, UK; 7 Division of Psychiatry, Centre for Clinical Brain Sciences, University of Edinburgh, Edinburgh, UK; 8 South London and Maudsley NHS Foundation Trust, London, UK

**Keywords:** Participation bias, Re-contact, GLAD Study, COPING study

## Abstract

**Background:**

The Genetic Links to Anxiety and Depression (GLAD) Study is a large cohort of individuals with lifetime anxiety and/or depression, designed to facilitate re-contact of participants for mental health research. At the start of the pandemic, participants from three cohorts, including the GLAD Study, were invited to join the COVID-19 Psychiatry and Neurological Genetics (COPING) study to monitor mental and neurological health. However, previous research suggests that participation in longitudinal studies follows a systematic, rather than random, process, which can ultimately bias results. Therefore, this study assessed participation biases following the re-contact of GLAD Study participants.

**Methods:**

In April 2020, all current GLAD Study participants (*N* = 36,770) were invited to the COPING study. Using logistic regression, we investigated whether sociodemographic, mental, and physical health characteristics were associated with participation in the COPING baseline survey (aim one). Subsequently, we used a zero-inflated negative binomial regression to examine whether these factors were also related to participation in the COPING follow-up surveys (aim two).

**Results:**

For aim one, older age, female gender identity, non-binary or self-defined gender identities, having one or more physical health disorders, and providing a saliva kit for the GLAD Study were associated with an increased odds of completing the COPING baseline survey. In contrast, lower educational attainment, Asian or Asian British ethnic identity, Black or Black British ethnic identity, higher alcohol consumption at the GLAD sign-up survey, and current or ex-smoking were associated with a reduced odds. For aim two, older age, female gender, and saliva kit provision were associated with greater COPING follow-up survey completion. Lower educational attainment, higher alcohol consumption at the GLAD Study sign-up, ex-smoking, and self-reported attention deficit hyperactivity disorder had negative relationships.

**Conclusions:**

Participation biases surrounding sociodemographic and physical health characteristics were particularly evident when re-contacting the GLAD Study volunteers. Factors associated with participation may vary depending on study design. Researchers should examine the barriers and mechanisms underlying participation bias in order to combat these issues and address recruitment biases in future studies.

**Supplementary Information:**

The online version contains supplementary material available at 10.1186/s12888-023-04890-x.

## Introduction

The Genetic Links to Anxiety and Depression (GLAD) Study is an online research project that recruits individuals who have experienced anxiety and/or depression, and enables the recontact and follow-up of enrolled participants [1]. Studies have already begun recruiting from this resource. This includes the COVID-19 Psychiatry and Neurological Genetics (COPING) study, a longitudinal study assessing mental health and well-being in response to and throughout the COVID-19 pandemic. In the COPING study, participants initially had the opportunity to complete a baseline survey, and could then consent to complete repeated follow-up surveys. However, whilst the full GLAD Study cohort was recontacted and invited to take part in COPING, just over a third of the participants completed the baseline questionnaire [2]. Since GLAD volunteers were given equal opportunity to take part in COPING, differences in participation could indicate self-selection bias in online surveys following re-contact.

Sociodemographic and health factors have repeatedly been linked with participation and attrition in longitudinal studies. Concerning sociodemographic characteristics, previous studies have found associations between increased participation in longitudinal research and older age [3–5], female sex [6, 7], self-identifying as White [3, 5, 8], being employed [4, 5, 9], being married [10, 11], and having higher levels of educational attainment [5, 12–14]. In contrast, decreased participation in longitudinal studies has been linked with greater levels of smoking [10, 14, 15], and varying levels of alcohol consumption [6, 10, 16]. Overall, these findings suggest that participation in research can follow a systematic rather than random process.

Despite extensive research linking sociodemographic characteristics to participation, there has been little theoretical work to explain the overall patterns of findings in the literature. Some researchers suggest that sociodemographic factors generally indicating greater social disadvantage are associated with reduced participation [15, 17, 18]. This is supported by several studies that have found lower levels of participation among those with lower educational attainment [3, 19], those identifying as belonging to an ethnic minority group [3, 8], and those who are unemployed [4]. This reduced participation may be indicative of barriers that hinder socially disadvantaged persons from volunteering in research, such as having less time to complete research tasks, being less able to sacrifice time for participation without financial compensation, and researchers predominantly recruiting participants from university settings. Further research is necessary to better understand the underlying mechanisms that contribute to the over- or under-representation of certain sociodemographic groups in mental health research. In particular, studies with large samples will enable multivariate analyses to ascertain which sociodemographic characteristics are independently associated with participation.

Beyond sociodemographic factors, mental and physical health characteristics have also been associated with participation bias. For instance, increases in the severity of anxiety and depressive symptoms, experiencing mental health disorders, and experiencing mental health comorbidities have been associated with reduced participation [3, 7, 11, 12, 14, 15, 19, 20]. This is illustrated by Knudsen et al.’s [20] study, which, by accessing non-participants' national registry information, found that persons who had been awarded a disability pension for mental health disorders had a threefold greater risk of nonparticipation compared to persons without a disability pension. The findings around mental health and participation parallel previous results surrounding physical health, where participants with more severe symptoms of poor health have demonstrated greater attrition [6, 8, 10, 16, 20–22]. Collectively, this would suggest that poorer psychiatric and/or physical health is associated with lower participation in research.

Nevertheless, other studies have failed to identify this link between poorer mental and physical health with reduced participation. Several mental health disorders and psychiatric symptoms have shown few or no associations with participation [4, 23, 24], or associations that have diminished over time [6]. Furthermore, another study found that having a chronic physical health disorder did not influence participation in a 3-year follow-up for a mental health study [4]. Several reasons could explain these conflicting results, such as different ways of assessing health characteristics and whether these previous studies were primarily recruiting participants to investigate physical or mental health. Further research simultaneously controlling for mental and physical health factors will help to clarify which characteristics are related to participation, and the underlying mechanisms driving their associations.

The primary aim of this study was to investigate whether the sociodemographic, mental health, and physical health characteristics of the GLAD Study cohort were associated with participation in the COPING baseline survey. This study then examined whether these factors were correlated with the overall number of longitudinal follow-up surveys that the GLAD Study participants completed for the COPING study. Hypotheses were stated in the pre-registration of this project, which can be found on the Open Science Framework: https://osf.io/gkxau. Given the large sample size of the GLAD Study cohort, these potential characteristics related to participation were investigated simultaneously to examine which factors were independently associated with participation. This investigation will help future researchers to understand potential participation patterns that could bias their sample and results when re-contacting participants from large mental health cohorts for recruitment. Such participation biases could then be more actively considered in research study design and analysis planning. Moreover, the findings will also expand on past research by including several factors that have rarely been investigated in relation to participation, such as non-binary or self-defined gender identities and the provision of genetic data.

## Methods

### Participants

The GLAD Study was launched in September 2018 to recruit a large, re-contactable sample of individuals with anxiety and/or depression to facilitate mental health research. The study was still ongoing at the time of publication. Participants have been recruited through a social media campaign and NHS organisations offering to support the study [1]. Volunteers have registered, consented, and completed an online questionnaire on the GLAD Study website (https://gladstudy.org.uk). Those who were eligible through screening were then sent a saliva kit through the post for DNA analysis. Eligibility criteria included being aged 16 or over, currently living in the UK, and either self-reporting a lifetime diagnosis of an anxiety or depressive disorder or meeting the Diagnostic and Statistical Manual-5 (DSM-5) criteria for one of these disorders. Ethical approval for the GLAD Study was obtained from the Research Ethics Committee on 21st August 2018 (REC reference: 18/LO/1218) following a full review by the committee. Davies et al. (2019) [1] provides a full description of the recruitment and data collection procedures for the GLAD Study. The COPING study was submitted separately for ethical review and approval was granted by the Research Ethics Committee (REC reference: 20/SW/0078).

**Fig. 1:**
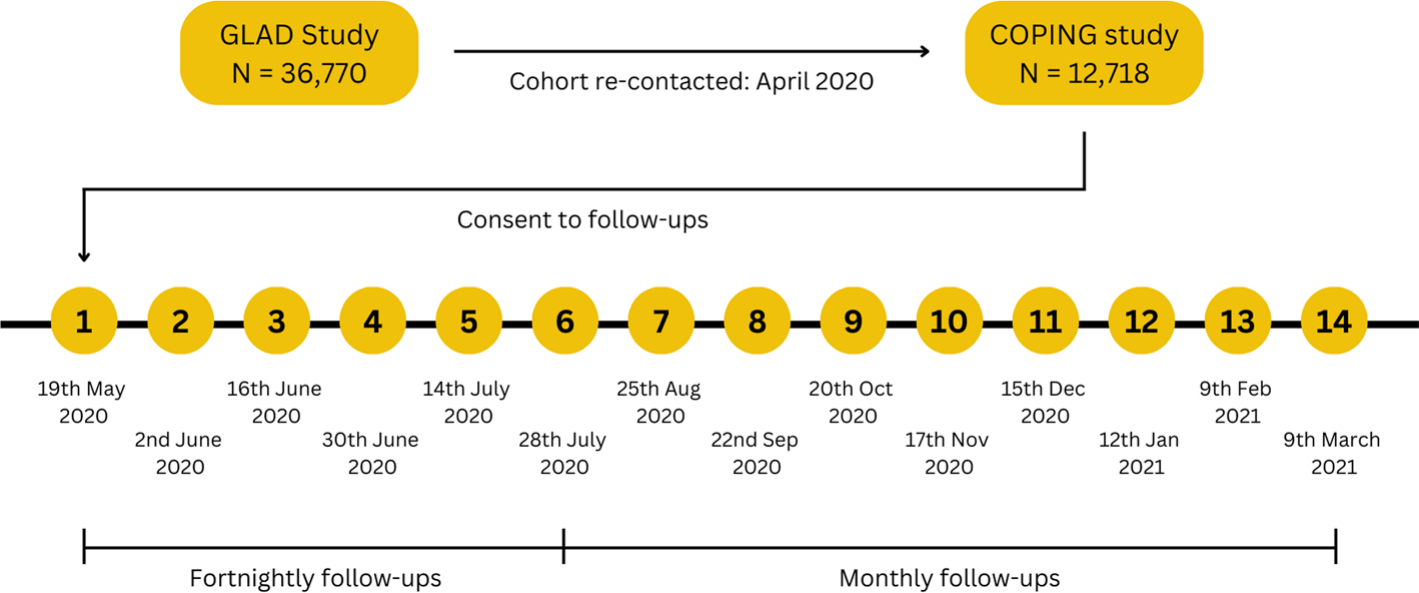
The COPING study timeline for re-contact and follow-ups of the GLAD Study cohort

The timeline for the COPING study is illustrated in Fig. 1 below. In April 2020, all GLAD Study participants that had completed the sign-up questionnaire at that time (*N* = 36,770) were emailed an invitation to take part in the COPING study. This resulted in 12,718 of the participants completing the baseline survey [2]. As part of the consenting process, participants were also provided with the opportunity to consent to further follow-up surveys, with the first survey sent on the 19th May 2020. The frequency of follow-ups changed from fortnightly to monthly at the sixth follow-up survey (28th July 2020), and to a three-monthly schedule at the 19th follow-up survey (27th July 2021). The present study utilises data from the COPING baseline survey and 14 out of 21 follow-up surveys.

It is worth noting that the COPING study also recruited volunteers by re-contacting participants from the National Institute for Health and Care Research (NIHR) BioResource (NBR) and Eating Disorders Genetics Initiative UK (EDGI UK) cohorts. Nevertheless, this study solely focused on the GLAD Study cohort because the investigators did not have access to baseline data from the NBR. Additionally, the COPING EDGI UK cohort was small and was therefore omitted to simplify the analyses to a single participant cohort.

### Measures

Data on sociodemographic, mental health, and physical health factors were assessed during the GLAD Study sign-up questionnaire and are described below. Genetic samples were returned by 24,133 of the participants. Additional information about variable recoding can be found in Additional file 1.

### Explanatory variables

The sociodemographic variables included age (continuous; measured in years), gender (categorical; recoded to 3 levels), ethnicity (categorical; 6 levels), highest educational attainment (categorical; 6 levels), employment (categorical; 7 levels), partnership status (categorical; recoded to 3 levels), and smoking (categorical; 3 levels).

The Alcohol Use Disorders Identification Test (AUDIT) is a 10-item scale which was used to assess hazardous and harmful alcohol consumption [25]. Each item in the scale has a range of 0–4, which are summed to create a total score between 0–40. Higher scores indicate more hazardous and harmful alcohol use.

The 9-item Patient Health Questionnaire (PHQ-9) is a self-report measure of current depressive symptoms [26]. Each item is a diagnostic symptom of major depressive disorder (MDD) and is rated on a 0 (not at all) to 3 (nearly every day) scale, producing a range of scores from 0 to 27. The overall PHQ-9 has a test–retest reliability of 0.84 and, using a cut-off score of ≥ 10, has a sensitivity of 88% and specificity of 88% for MDD [26].

The 7-item Generalised Anxiety Disorder Assessment (GAD-7) is a self-report measure for current anxiety symptoms [27]. Each item is rated on a 0 (not at all) to 3 (nearly every day) scale, producing a range of scores from 0 to 21. The GAD-7 has a test–retest reliability of 0.83 and, using a GAD-7 cut-off score of ≥ 10, has a sensitivity of 89% and a specificity of 82% for GAD [27].

Mental health diagnoses were captured via a single-item self-report question assessing whether the participant had been diagnosed with a focal mental health disorder by a clinician during their lifetime. The participants’ responses were combined to create the following diagnostic categories: i) depression and anxiety, ii) depression only, iii) anxiety only, iv) no depression or anxiety, v) eating disorders, vi) obsessive compulsive disorders, vii) psychotic and bipolar disorders, viii) psychotic disorder only, ix) bipolar disorder only, x) post-traumatic stress disorder, xi) autism spectrum disorders, xii) attention deficit hyperactivity disorder, and xiii) personality disorder. More details about how each of the individual diagnoses were categorised can be found in Additional file 1. A count of the participants’ total mental health disorder diagnoses was also calculated from these self-reported diagnoses.

Participants self-reported physical health conditions by responding yes or no to prior diagnoses of: asthma, emphysema or chronic bronchitis, heart attack or angina, cancer (breast, lung, stomach, colon, uterus, prostate), epilepsy or convulsions, diabetes type I and II, high blood pressure, high blood cholesterol, stroke, and migraines. Responses were then used to derive a categorical physical health comorbidity variable representing participants with zero, one, or two or more of the above conditions.

In the GLAD Study, once the participants complete the online sign-up questionnaire, they are sent a saliva kit to sample their genetic data, to be returned by post. A binary variable was created to reflect whether the participant had returned their saliva kit or not.

### Outcome variables

Participation in the COPING baseline survey was represented by a derived binary variable that categorised participants as having completed the survey or not. By contrast, participation in the follow-up surveys was assessed by deriving a count variable reflecting how many surveys each participant had completed (0–14). For both outcome variables, a completed survey was defined as reaching the end of the survey regardless of the amount of missing data throughout the response.

### Statistical analyses

All the analyses were conducted with R, version 4.1.2 2021–11-01 [28]. Correlations between all the exploratory and outcome variables were conducted to assess for multicollinearity using the Stats package, version 4.1.2 [28]. Histograms and boxplots were generated to check for outliers. Box-Tidwell tests and scatter plots were run for the continuous variables to test the linearity assumption for the logistic regression model.

To address the first aim, a binomial logistic regression model was carried out using the glm function from the Stats package.Sociodemographic, physical, and mental health characteristics were entered simultaneously to assess which factors were associated with participation in the COPING baseline survey.

For the secondary aim, a Zero-Inflated Negative Binomial (ZINB) regression was conducted to examine which of the aforementioned factors were associated with the number of completed COPING follow-up surveys. This was done using the zeroinfl function from the pscl package, version 1.5.5 [29]. The ZINB regression model broadly adjusts for excess zeros appearing in a data set, such as a large proportion of participants in the COPING study not completing any follow-up surveys. It achieves this by calculating a count model, which conducts a negative binomial regression for the outcome, and a zero model, which involves a logistic regression to compare zero and non-zero responses.

Bonferroni adjustment was used to correct for multiple testing of all the models in this study. This involves dividing the conventional *p*-value (0.05) by the number of tests being undertaken [30]. Scripts for these analyses are available for open access at https://github.com/StevenJBright/COPING_participation.

## Results

**Fig. 2:**
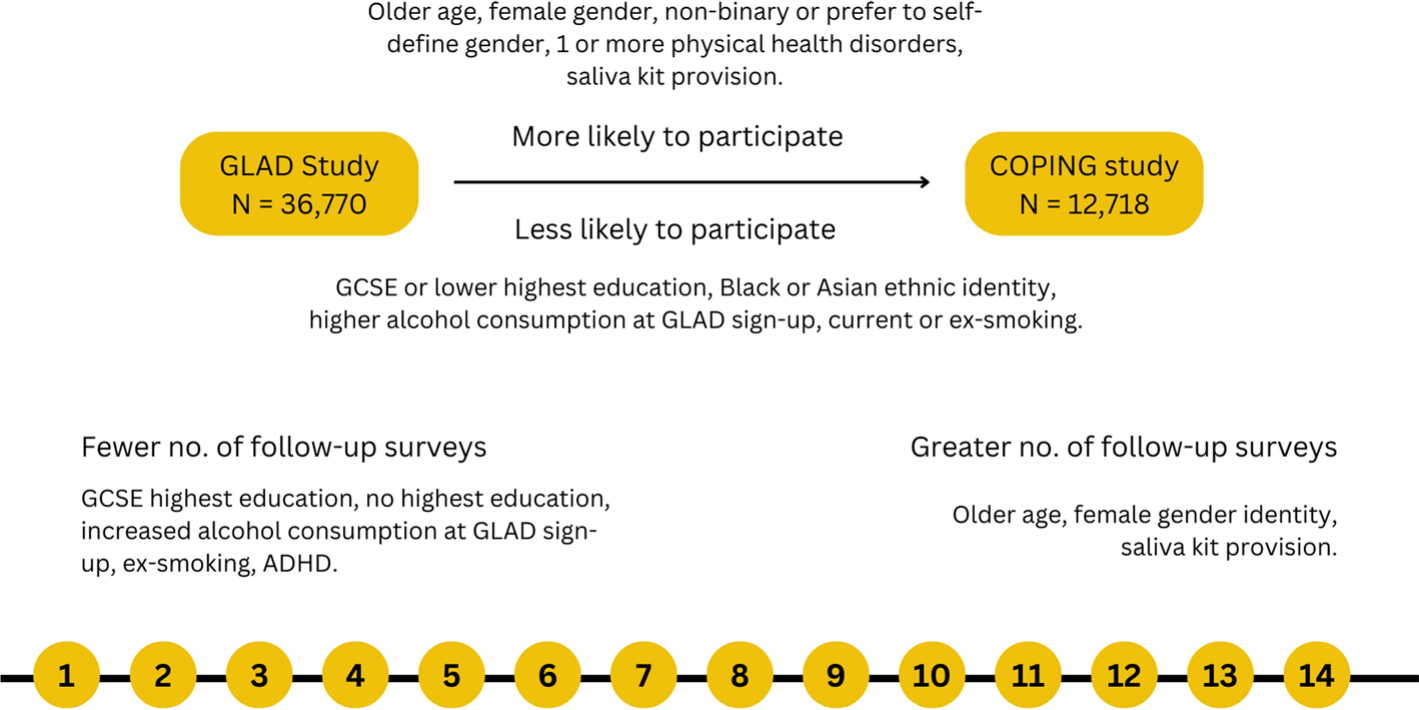
Factors associated with participation across the COPING baseline and follow-up surveys after multiple testing corrections

Figure 2 summarises the main characteristics significantly associated with participation in the COPING baseline (aim one) and follow-up surveys (aim two). The results from each of these aims are subsequently reported.

Results from the correlation analyses found no evidence of multicollinearity. The boxplots indicated that some observations on the age, AUDIT, and total number of self-reported mental health disorder variables could be outliers. However, when inspecting the histograms, the observed values were plausible and were a natural continuation of the distribution of the variables’ values. As a result, the observations were not modified or excluded from the analyses.

Box-Tidwell tests and scatter plots demonstrated that four variables violated the logistic regression linearity assumption. A sensitivity analysis was conducted whereby this logistic regression model was re-ran with these variables categorised as factors. The results of this sensitivity analysis showed that, after Bonferonni adjustment, all variables that were originally significant in the aim 1 logistic regression model remained significant with the same effect size direction. Notably, when the AUDIT sum score was treated as a factor, its highest level of possible dependence was significant and this was thus likely driving the association when it was treated as a continuous variable. Further information about these analyses and the full model results can be found in Additional files 1 and 2, respectively.

### Factors associated with participation in the COPING baseline survey

A logistic regression model was conducted to assess the sociodemographic, mental and physical health factors associated with participation in the COPING baseline survey. Results from this model are summarised in Table 1.

**Table 1:** Sociodemographic, mental health and physical health predictors of participation in the COPING baseline survey

Variable	Did not complete COPING baseline (*N* = 21,802)	Completed COPING baseline (*N* = 12,483)	OR (CI)
Intercept			0.01 (0—0.26)
Age	36 (13.82)	42.2 (14.61)	1.03 (1.03—1.03)
Gender (ref: Male)			
Female	16,583 (76%)	10,008 (80%)	1.59 (1.49—1.71)
Non-binary/Prefer not to say	457 (2.1%)	244 (2%)	1.68 (1.38—2.04)
Highest education (ref: College or university degree)			
A-levels/AS levels or equivalent	5,169 (24%)	2,490 (20%)	0.91 (0.85—0.97)
O-levels/GCSEs or CSEs or equivalent	4,163 (19%)	1,967 (16%)	0.7 (0.65—0.76)
NVQ, HND, HNC or equivalent	576 (2.6%)	284 (3.1%)	0.73 (0.61—0.86)
None of the above	923 (4.2%)	385 (3.1%)	0.55 (0.47—0.63)
Ethnicity (ref: White)			
Mixed	570 (2.6%)	273 (2.2%)	1.03 (0.87—1.22)
Asian or Asian British	353 (1.6%)	96 (0.8%)	0.58 (0.44—0.77)
Black or Black British	132 (0.6%)	37 (0.3%)	0.46 (0.29—0.7)
Arab	24 (0.1%)	7 (< 0.1%)	0.37 (0.08—1.16)
Other	212 (1%)	115 (0.9%)	0.85 (0.65—1.1)
Employment status (ref: Employed or self-employed)			
Retired	845 (3.9%)	1,154 (9.3%)	0.97 (0.85—1.09)
Looking after home and/or family	777 (3.6%)	416 (3.3%)	0.93 (0.81—1.07)
Unable to work because of sickness or disability	2,636 (12%)	1,468 (12%)	0.96 (0.88—1.05)
Unemployed	925 (4.3%)	415 (3.3%)	0.98 (0.85—1.13)
Doing unpaid or voluntary work	331 (1.5%)	234 (1.9%)	0.91 (0.74—1.1)
Full or part-time student	3,243 (15%)	1,176 (9.4%)	0.95 (0.86—1.04)
None of the above	174 (0.8%)	108 (0.9%)	0.92 (0.68—1.23)
Relationship status (ref: Single)			
Relationship or married/civil partnership	13,336 (62%)	7,957 (64%)	1 (0.94—1.07)
Divorced/widowed/separated	1,695 (7.9%)	1,351 (11%)	0.94 (0.85—1.04)
PHQ	12.4 (6.97)	11.2 (6.86)	1 (1—1.01)
GAD	10 (6.07)	8.8 (6.06)	0.99 (0.99—1)
AUDIT	7.4 (6.74)	6.3 (6.02)	0.99 (0.98—0.99)
Smoking status (ref: Never smoked)			
I smoke now	4,530 (21%)	1,502 (12%)	0.74 (0.68—0.8)
I used to smoke	6,726 (31%)	4,206 (34%)	0.88 (0.83—0.93)
Physical health comorbidities (ref: No physical health disorders)			
1 physical health disorder	7,517 (35%)	4,480 (36%)	1.11 (1.05—1.18)
2 + physical health disorders	3,956 (18%)	2,952 (24%)	1.19 (1.1—1.27)
Total mental health disorders	2.6 (1.11)	2.5 (1.08)	1.06 (0.98—1.14)
Anxiety and depression comorbidity (ref: Anxiety and depressive disorder)			
No anxiety or depressive disorder	147 (0.8%)	82 (0.8%)	0.83 (0.59—1.15)
Depressive disorder only	1,892 (10%)	1,328 (12%)	1.07 (0.95—1.2)
Anxiety disorder only	339 (1.8%)	223 (2.1%)	0.94 (0.77—1.15)
Eating disorders (ref: No eating disorder)	2,520 (12%)	1,370 (11%)	0.94 (0.84—1.05)
Obsessive–compulsive related disorders (ref: No OCRDs)	2,813 (13%)	1,397 (11%)	0.89 (0.8—1)
Psychotic and bipolar disorders (ref: No psychotic and bipolar disorder)	295 (1.4%)	163 (1.3%)	0.85 (0.65—1.12)
Only psychotic disorder	453 (2.1%)	229 (1.8%)	0.89 (0.73—1.08)
Only bipolar disorder	1,275 (5.9%)	692 (5.5%)	0.87 (0.76—0.99)
Autistic spectrum disorder (ref: No ASD)	732 (3.4%)	378 (3%)	1.15 (0.97—1.36)
Attention Deficit (/Hyperactivity) Disorder (ref: No ADHD or ADD)	551 (2.5%)	199 (1.6%)	0.76 (0.62—0.93)
Personality disorder (ref: No PD)	1,823 (8.4%)	932 (7.5%)	1.04 (0.92—1.18)
Start date			1 (1—1)
Saliva kit returned (ref: No saliva kit provided)	13,362 (61%)	10,771 (86%)	3.6 (3.38—3.85)

This table displays results from the logistic regression model examining the relationship between sociodemographic, mental health and physical health factors with participation in the COPING follow-up surveys. A variable with an OR > 1 indicates that participation in the COPING baseline survey become more likely as the characteristic increases, whereas an OR of < 1 indicates that participation becomes less likely as the characteristic increases. The bonferroni adjusted *p*-value threshold was 0.00119 (0.05 / 42), and an asterisk indicates significance at this threshold

*Abbreviations: PHQ* Patient Health Questionnaire-9, *GAD* Generalised Anxiety Disorder Assessment 7, *AUDIT* Alcohol Use Disorders Identification Test, *OCRD* Obsessive–Compulsive Related Disorder, *ASD* Autistic Spectrum Disorder, *AD(H)D* Attention Deficit Hyperactivity Disorder, *PD* Personality Disorder

The following characteristics were associated with a significantly *increased* odds of participation in the COPING baseline survey: older age, female gender identity (ref: male), non-binary or prefer to self-define gender identity (ref: male), having one, two or more physical health disorders (ref: no physical health disorders), and saliva kit provision (ref: no saliva kit provision). By contrast, the following factors were associated with a *reduced* odds of participation: A-level or lower educational attainment (ref: college or university degree), Asian or Asian British ethnic identity (ref: White), Black or Black British ethnic identity (ref: White), experiencing higher anxiety symptoms and greater alcohol consumption at the GLAD sign-up survey, being a current or ex-smoker (ref: never smoked), and having a self-reported diagnosis of obsessive–compulsive or related disorders (OCRDs; ref: no OCRDs), bipolar disorder (ref: no bipolar disorder), or attention deficit hyperactivity disorder (ADHD; ref: no ADHD). After adjusting the *p*-value threshold for multiple testing, anxiety symptoms, OCRDs, bipolar disorder, and ADHD became non-significant.

### Factors associated with participation in the COPING follow-up surveys

**Fig. 3:**
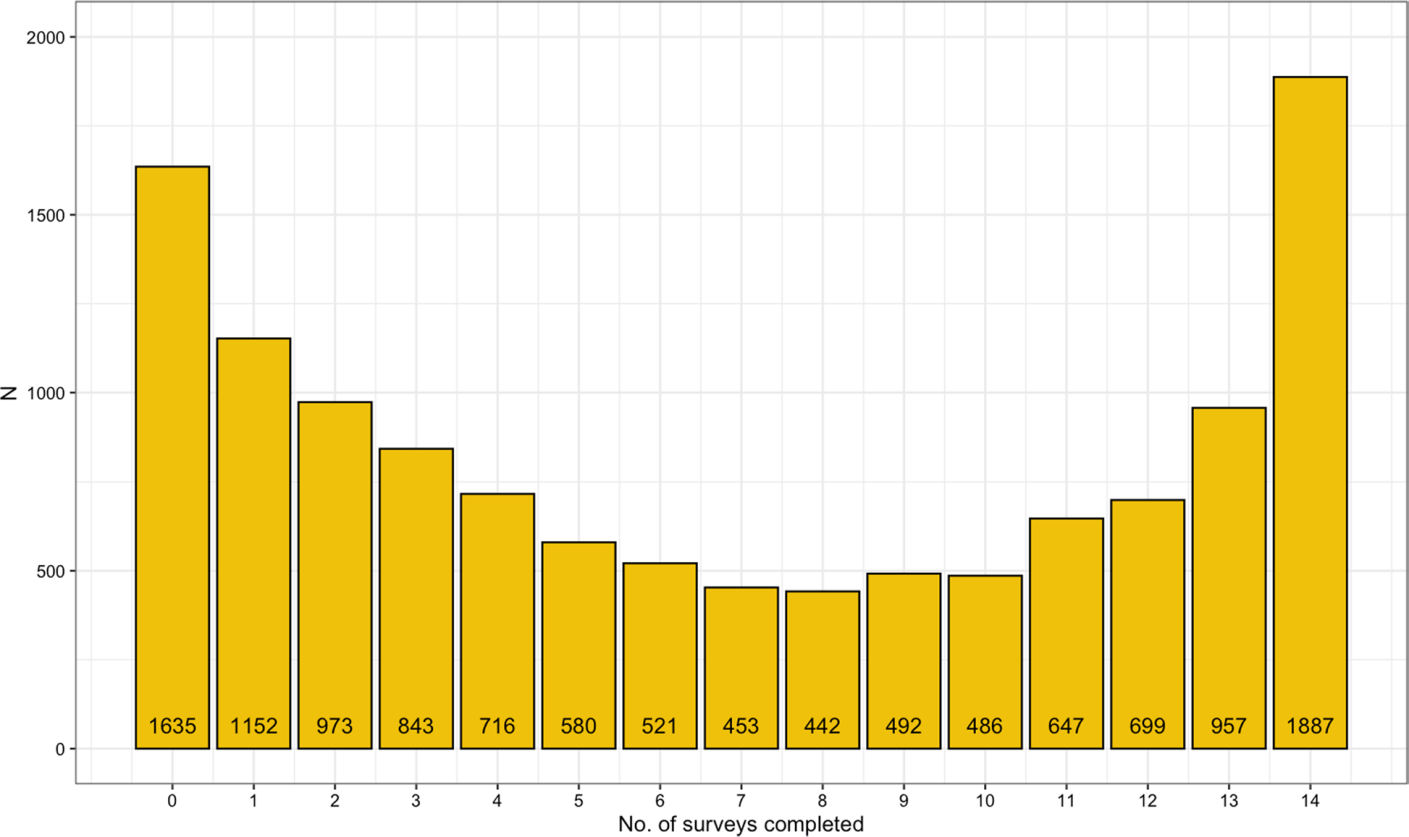
Total COPING follow-up surveys completed by GLAD Study volunteers who completed the COPING baseline survey

A ZINB regression model was used to examine participation biases in the COPING follow-up surveys. As illustrated in Fig. 3 below, many participants either completed a small number *or* the vast majority of the COPING follow-up surveys. Additionally, an appreciable number of participants did not complete any follow-up surveys after completing the COPING baseline survey.

The results of the ZINB model are summarised in Table 2, in two parts. The first is the negative binomial regression, which results in a rate ratio representing the likelihood of participating in a greater number of COPING follow-up surveys. Older age, female gender (ref: male), and saliva kit provision (ref: no saliva kit provision) were associated with an increased likelihood of completing more COPING follow-up surveys. By contrast, the following variables were associated with a decreased likelihood of completing more follow-up surveys: GCSE highest educational attainment or having none of the specified educational qualifications (ref: college or university degree), increased alcohol consumption at the GLAD sign-up survey, being an ex-smoker (ref: never smoked), and self-reporting a diagnosis of ADHD (ref: no ADHD). These characteristics were all significant at the adjusted *p*-value threshold.

**Table 2:** Negative binomial and Zero-inflated negative binomial model of participation in the COPING study follow-up surveys

	**Negative binomial regression**		**Zero-inflated model**	
**Explanatory variable**	Rate ratio	95% CI	Rate ratio	95% CI
Intercept	3.68*	3.26—4.17	0.63	0.34—1.15
Age	1.01*	1.01—1.02	0.96*	0.95—0.97
Female	1.07*	1.03—1.11	0.8	0.65—0.99
Non-binary/Prefer not to say	1.07	0.95—1.19	0.59	0.33—1.05
A-levels/AS levels or equivalent	0.97	0.93—1	1.1	0.91—1.32
O-levels/GCSEs or CSEs or equivalent	0.9*	0.86—0.94	1.41*	1.14—1.73
NVQ, HND, HNC or equivalent	0.88	0.8—0.97	1.27	0.79—2.06
None of the above	0.88*	0.81—0.95	0.9	0.54—1.5
Mixed ethnicity	0.96	0.87—1.07	1.43	0.97—2.12
Asian or Asian British	1.05	0.87—1.27	1.96	1.06—3.65
Black or Black British	0.82	0.62—1.09	0.6	0.1—3.41
Arab	0.77	0.35—1.69	0	0—5.4249486520193E + 271
Other	0.96	0.83—1.12	0.57	0.2—1.63
Retired	1.04	0.98—1.09	1.09	0.73—1.64
Looking after home and/or family	0.98	0.91—1.06	0.58	0.35—0.97
Unable to work because of sickness or disability	1.04	0.99—1.1	1.07	0.84—1.36
Unemployed	1.06	0.98—1.15	0.89	0.6—1.32
Doing unpaid or voluntary work	1.05	0.94—1.16	0.69	0.34—1.37
Full or part-time student	0.95	0.89—1.01	0.97	0.75—1.24
None of the above	1.01	0.86—1.18	0.84	0.32—2.24
Relationship or married/civil partnership	0.97	0.94—1.01	0.97	0.82—1.15
Divorced/widowed/separated	0.97	0.92—1.02	1.04	0.76—1.41
PHQ	1	1—1	1	0.98—1.02
GAD	0.99	0.99—1	1.02	1—1.03
AUDIT	1*	0.99—1	1	0.99—1.01
I smoke now	0.93	0.89—0.98	1.38	1.11—1.7
I used to smoke	0.93*	0.9—0.96	1.18	0.99—1.4
1 physical health disorder	1.02	0.98—1.05	0.99	0.84—1.16
2 + physical health disorders	0.98	0.95—1.02	0.86	0.7—1.06
Mental health comorbidities	0.99	0.95—1.03	1.06	0.86—1.29
No depressive or anxiety disorder	1.07	0.9—1.29	1.73	0.7—4.28
Depressive disorder only	1.01	0.95—1.08	1.12	0.81—1.54
Anxiety disorder only	1.01	0.91—1.13	1.64	0.97—2.77
Eating disorders	1.01	0.95—1.08	0.84	0.61—1.15
OCRDs	0.97	0.91—1.03	0.93	0.68—1.27
Psychotic and bipolar disorder	0.98	0.84—1.14	0.6	0.25—1.47
Only psychotic disorder	1.04	0.93—1.16	0.7	0.37—1.31
Only bipolar disorder	1.01	0.93—1.09	1.16	0.81—1.66
ASD	1.1	1—1.21	0.75	0.46—1.2
ADHD or ADD	0.77*	0.67—0.87	0.6	0.28—1.27
Personality disorder	1.05	0.98—1.13	1.11	0.79—1.56
Saliva kit	1.22*	1.17—1.28	0.71*	0.59—0.86

This table displays results from the zero-inflated negative binomial regression examining the relationship between sociodemographic, mental health and physical health factors with participation in the COPING follow-up surveys. The bonferroni adjusted *p*-value threshold was 0.00122 (0.05 / 41), and an asterisk indicates significance at this threshold

*Abbreviations: PHQ* Patient Health Questionnaire-9, *GAD* Generalised Anxiety Disorder Assessment 7, *AUDIT* Alcohol Use Disorders Identification Test, *OCRD* Obsessive–Compulsive Related Disorder, *ASD* Autistic Spectrum Disorder, *AD(H)D* Attention Deficit Hyperactivity Disorder

The zero-inflated part of the model assesses associations with the likelihood of *not* completing a COPING follow-up survey. Hence, rate ratio values > 1 represent an increased likelihood of *not* participating in COPING follow-up, and values < 1 represent an increased likelihood of participation. For example, the rate ratio for age is 0.96, indicating that younger participants are more likely to *not* have completed any COPING follow-ups, whereas as age increases so does the likelihood of having taken part in the follow-ups. Older age and saliva kit provision were associated with a *decreased* likelihood of not having completed a follow-up survey (i.e., more likely to have taken part in the follow-ups). In contrast, GCSE highest educational attainment was associated with an *increased* likelihood of not having completed a follow-up survey (i.e., less likely to have taken part in the follow-ups). These variables were all significant at the adjusted *p*-value threshold.

### Exploratory analyses

Results from the models demonstrated a large effect of saliva kit provision. When interpreting these findings, it was noted that this variable is itself a measure of participation in the GLAD Study. Therefore, both the logistic and ZINB regression models were re-run with only volunteers who had returned a saliva kit to examine whether these characteristics associated with participation in the COPING baseline and follow-up surveys differed from the full sample. Results from these exploratory analyses, including the primary aim models, are available in the Additional file 2.

In the exploratory logistic regression model, the majority of the factors associated with completing the COPING baseline survey amongst the kit returners were the same as the full sample. The only difference was that having a Black or Black British ethnic identity was not significantly associated with completing the baseline survey, in contrast to the full sample. Similarly for the exploratory ZINB model, most of the same characteristics were associated with participation in the COPING follow-up surveys. However, in the count part of the exploratory model, having none of the available educational qualifications was no longer significantly associated with completing follow-up surveys compared to the full sample. In contrast, in the zero part of the exploratory model, ex-smoking was no longer significantly associated with participation in the follow-up surveys.

A further exploratory analysis was then conducted to examine factors associated with saliva kit provision in the GLAD Study. This was achieved by simultaneously entering the original sociodemographic, physical and mental health characteristics into a multiple logistic regression model, with saliva kit provision as the binary outcome. At the adjusted *p*-value threshold, the following variables were associated with an *increased* odds of providing a saliva kit: older age, being retired, being unable to work due to sickness or disability, being unemployed, doing unpaid or voluntary work, and being a full-time or part-time student (ref for all non-age variables: employed or self-employed). In contrast, the following variables were associated with a *decreased* odds of providing a saliva kit: female gender identity, A-levels or lower educational attainment, being married or in a civil partnership, being widowed, divorced or separated (partnership variables’ ref: single), increased anxiety and depressive symptoms at the GLAD Study sign-up, current smoking, ex-smoking, and having more than one physical health disorder.

## Discussion

The current study examined whether participation in the COPING study was associated with sociodemographic, mental health and physical health characteristics of the volunteers re-contacted from the GLAD Study. Assessing for potential participation biases in the COPING study invites future researchers recruiting from the GLAD Study, or other large-scale mental health cohorts, to consider the influence of participation bias on research and findings. The present study also benefits the wider scientific community by scrutinising prior findings and assessing previously unexplored characteristics that may be related to participation.

Many of the same characteristics were independently associated with participation in the COPING baseline and follow-up surveys after controlling for all factors under investigation. For instance, older age, identifying as female, and providing a saliva kit for the GLAD Study were broadly associated with increased participation across the COPING study. Aside from the provision of genetic data, which was unique to this study, these findings are consistent with previous studies investigating participation in longitudinal research [3, 6, 7, 14, 15]. In contrast, having a highest educational attainment of GCSEs or none of the specified educational qualifications, previous smoking, and higher alcohol consumption at the time of the GLAD Study sign-up were negatively associated with participation across the COPING study. These patterns of associations are also comparable to previous research, which shows that lower educational attainment [3], smoking [6, 16] and higher alcohol consumption were related to reduced participation [6, 10, 16].

There were, however, some differences between the factors associated with participation in the COPING baseline and follow-up surveys. Notably, a broader range of factors was associated with participation bias in the baseline survey. For example, having one or more physical health disorders was associated with increased participation in the baseline, but not follow-up surveys. This contradicts previous research that suggests individuals with poorer physical health have lower levels of participation [6, 8, 10, 16, 21, 22]. In contrast, having a Black or Asian ethnic identity or a non-binary or self-defined gender identity were associated with reduced participation in the COPING baseline survey only. Since these ethnic groups are minorities in the UK, this seems to contradict previous findings suggesting that people from ethnic minority groups broadly show lower levels of participation in research [3, 5, 8]. Instead, it appears that there is an initial barrier to participate, but for those who are willing to complete the baseline survey, there is no association with their long-term engagement. On the other hand, to our knowledge, a non-binary or self-defined gender identity has not previously been investigated in relation to participation bias. Finally, it is interesting that ADHD was only associated with reduced participation in the COPING follow-up surveys. Past research has shown that higher polygenic risk for ADHD is negatively associated with participation in longitudinal research [14, 15], and one may have expected this to be broadly associated with less participation in mental health research.

Several conclusions can be drawn from the findings of this study. Firstly, in line with previous literature, the GLAD Study volunteers’ participation after re-contact seems to follow a systematic, rather than random, process. Secondly, the results suggest that sociodemographic, physical health, and saliva kit provision are the factors most strongly associated with participation bias when re-contacting GLAD Study volunteers. In contrast, other potential characteristics that have demonstrated relationships with participation in past research, including mental health factors [3, 7, 11, 12, 14, 15, 19, 20], employment status [4, 5, 9], and partnership status [10, 11] were weakly or not significantly related to participation following re-contact. It is possible that these characteristics may be unrelated to participation after controlling for other factors. However, these findings need to be interpreted with caution, since GLAD is a mental health cohort composed of volunteers with a generally severe presentation of anxiety and/or depression [1]. For example, the UK Household Longitudinal Study (UKHLS) found that subjective health and employment status were associated with attrition in their general population sample [5]; however, our study found the opposite direction of effect for physical health disorders and no significant association for employment status. Some of the observed participation biases in this study may therefore only be generalisable to other large-scale mental health cohorts, or studies recruiting from GLAD.. It is likely that predictors of participation vary depending on the study aims, design, and sample population. We would therefore recommend that all studies consider what potential biases may impact participation and consider ways of addressing these in the study design. Researchers utilising recontact or longitudinal study design should additionally assess and report predictors of attrition.

Saliva kit provision was the strongest predictor of participation in COPING baseline and follow-up surveys. This may be a pertinent finding for researchers interested in re-contacting mental health cohorts whose participants have provided genetic data, such as the GLAD Study. This finding may be because providing a saliva kit is an element of participation in the GLAD Study, with participants returning a kit thereby showing a higher level of commitment to participate or to research more broadly. Furthermore, in the exploratory analyses, several characteristics were associated with saliva kit provision itself. For example, older age and being a student were associated with an increased odds of saliva kit provision, whereas educational attainment of A-levels or lower and current or previous smoking were related to a decreased odds of provision. Previous studies have similarly found participation biases surrounding genetic data provision, such as provision increasing amongst persons coming from a higher socioeconomic status and those with a greater familial risk of schizophrenia [22]. In contrast, several psychiatric diagnoses have predominantly shown negative associations with the provision of genetic data [31], and the representation of minoritised ethnic groups has also historically been an issue for genetic research studies (e.g., [32]). Overall, researchers should consequently be mindful of participation bias when collecting genetic data or re-contacting volunteers in contexts where genetic data provision is relevant.

It is noteworthy that there were some differences in the factors associated with saliva kit provision compared to participation in COPING. For example, employment and partnership statuses were only associated with saliva kit provision. Furthermore, while having physical health disorders and female gender identity were associated with increased participation in COPING, both characteristics were associated with reduced odds of saliva kit provision. Collectively, this suggests that the characteristics associated with participation, and the direction of their relationship, may vary according to the form of participation.

This study supports previous recommendations to actively consider participation bias in research, such as by oversampling groups of volunteers that are associated with lower levels of participation [19], and conducting sensitivity analyses [31]. Such considerations could help to mitigate the negative consequences surrounding participation bias, such as the sample representativeness [3] and erroneous relationships between variables [33, 34].

Several limitations should be considered when interpreting these findings. Firstly, participation in the COPING study was defined as reaching the end of the survey, regardless of the amount of missing data. This overlooks the potential nuances of characteristics associated with different levels of missingness [4], such as full responders with no missing data compared to partial responders, which could be examined in future research. Secondly, the COPING study was conducted entirely online and only involved completing surveys. Therefore, this study’s results may not represent participation biases impacting other types of research, such as in-person studies or clinical trials [19]. Thirdly, this study investigated participation biases in a mental health-orientated, COVID-19 study during a global pandemic, which involved nationwide experiences, such as rising unemployment and national lockdowns [35]. Consequently, some of the observed associations in this study may not generalise to participation biases in longitudinal health research outside the pandemic. Fourthly, the GLAD Study predominantly utilises online recruitment methods [1]. Therefore, the participation biases observed in this study may only relate to a specific group of volunteers, such as people who are enthusiastic about research and who can access the internet [36]. Finally, certain populations were underrepresented, such as people from ethnic minority backgrounds. As a result, these findings may not generalise to studies recruiting from the general population.

There are several future directions for investigations of participation biases in research. Firstly, researchers with data throughout the COVID-19 pandemic could examine whether fluctuations in sociodemographic or health characteristics, such as mental health symptoms and employment status, are related to changes in study participation throughout the pandemic. This was beyond the scope of the current study, which solely utilised pre-pandemic data from the GLAD Study sign-up survey. Secondly, future studies could replicate and/or extend our exploratory investigation into factors associated with the provision of genetic data. This would be useful because our exploratory analyses did show some discrepancies with the factors associated with genetic data provision compared to past research (e.g. [31]). Finally, although researchers could attempt to correct for bias through techniques, such as oversampling underrepresented groups [19] or survey weights [5, 37], we are cautious about recommending this approach as it does not address inherent biases that may impact participation (i.e., people from underrepresented groups who took part in the study may vary in other ways from the general population, e.g., higher prosocial behaviour). We therefore recommend instead that future researchers more closely examine the barriers and mechanisms underlying the associations between certain characteristics and participation, enabling researchers to combat these issues and address recruitment biases in future research.

## Conclusions

Overall, this study broadly supports previous research on participation bias by showing that the GLAD volunteers’ participation in the COPING study followed a systematic, non-random process. In particular, participation was associated with older age, identifying as female, having a non-binary or prefer to self-define gender identity, and providing a saliva kit. By contrast, GCSE or lower educational attainment, identifying as Asian or Black, current or previous smoking, having physical health disorders, and self-reported ADHD were associated with decreased participation. This study has implications for future research recruiting from large-scale mental health cohorts, suggesting that participation bias could undermine the representativeness of the sample and impact results. Further research is needed to help to clarify which characteristics are independently associated with participation bias, the mechanisms for these associations, and how they can be addressed.

## Supplementary Information

**Additional file 1:** Further methodological information and variable correlation results.

**Additional file 2.** Output from the statistical models.

## Data Availability

The GLAD Study and COPING study datasets that were used and analysed during the current study are available from the corresponding author on reasonable request. All of the code for cleaning and analysing the data are available online at: https://github.com/StevenJBright/COPING_participation.

## Abbreviations

AUDIT: Alcohol Use Disorders Identification Test
ADHD: Attention Deficit Hyperactivity Disorder
COPING study: COVID-19 Psychiatry and Neurological Genetics study
DSM-5: Diagnostic and Statistical Manual-5
EDGI: Eating Disorders Genetics Initiative
GCSE: General Certificate of Secondary Education
GAD-7: Generalised Anxiety Disorder Assessment
GLAD Study: Genetic Links to Anxiety and Depression Study
MDD: Major Depressive Disorder
NIHR: National Institute for Health and Care Research;
NBR: National BioResource
OCRDs: Obsessive-Compulsive or Related Disorders
PHQ-9: Patient Health Questionnaire
REC: Research Ethics Committee
ZINB: Zero-Inflated Negative Binomial regression

## References

[1] Davies MR, Kalsi G, Armour C, Jones IR, McIntosh AM, Smith DJ (2019). The Genetic Links to Anxiety and Depression (GLAD) Study: Online recruitment into the largest recontactable study of depression and anxiety. Behav Res Ther.

[2] Young KS, Purves KL, Hübel C, Davies MR, Thompson KN, Bristow S, et al. Depression, anxiety and PTSD symptoms before and during the COVID-19 pandemic in the UK. 2021. Available from: psyarxiv.com/sf7b6 10.1017/S0033291722002501 PMC10482709 35879886

[3] Czeisler MÉ, Wiley JF, Czeisler CA, Rajaratnam SMW, Howard ME (2021). Uncovering survivorship bias in longitudinal mental health surveys during the COVID-19 pandemic. Epidemiol Psychiatr Sci.

[4] de Graaf R, van Dorsselaer S, Tuithof M, ten Have M (2013). Sociodemographic and psychiatric predictors of attrition in a prospective psychiatric epidemiological study among the general population. Result of the Netherlands Mental Health Survey and Incidence Study-2. Compr Psychiatry..

[5] Cabrera Alvarez P, James N Lynn P. Panel attrition in the General Population Sample and the Immigrant and Ethnic Minority Boost of Understanding Society. Understanding Society Working Paper. 2023: Colchester: University of Essex.

[6] Bellón JA, de Dios LJ, Moreno B, Montón-Franco C, GildeGómez-Barragán MJ, Sánchez-Celaya M (2010). Psychosocial and sociodemographic predictors of attrition in a longitudinal study of major depression in primary care: the predictD-Spain study. J Epidemiol Community Health.

[7] Kekkonen V, Kivimäki P, Valtonen H, Hintikka J, Tolmunen T, Lehto SM (2015). Sample selection may bias the outcome of an adolescent mental health survey: results from a five-year follow-up of 4171 adolescents. Public Health.

[8] Radler BT, Ryff CD (2010). Who participates? Accounting for longitudinal retention in the MIDUS national study of health and well-being. J Aging Health.

[9] Cheng A, Zamarro G, Orriens B (2020). Personality as a Predictor of Unit Nonresponse in an Internet Panel. Sociol Methods Res.

[10] Torvik FA, Rognmo K, Tambs K (2012). Alcohol use and mental distress as predictors of non-response in a general population health survey: the HUNT study. Soc Psychiatry Psychiatr Epidemiol.

[11] Young AF, Powers JR, Bell SL (2006). Attrition in longitudinal studies: who do you lose?. Aust N Z J Public Health.

[12] Frojd SA, Kaltiala-Heino R, Marttunen MJ (2011). Does problem behaviour affect attrition from a cohort study on adolescent mental health?. Eur J Public Health.

[13] Hansson I, Berg AI, Thorvaldsson V (2018). Can personality predict longitudinal study attrition? Evidence from a population-based sample of older adults. J Res Pers.

[14] Taylor AE, Jones HJ, Sallis H, Euesden J, Stergiakouli E, Davies NM (2018). Exploring the association of genetic factors with participation in the Avon Longitudinal Study of Parents and Children. Int J Epidemiol.

[15] Cornish RP, Macleod J, Boyd A, Tilling K (2021). Factors associated with participation over time in the Avon Longitudinal Study of Parents and Children: a study using linked education and primary care data. Int J Epidemiol.

[16] May AM, Adema LE, Romaguera D, Vergnaud AC, Agudo A, Ekelund U (2012). Determinants of non- response to a second assessment of lifestyle factors and body weight in the EPIC-PANACEA study. BMC Med Res Methodol.

[17] Howe LD, Tilling K, Galobardes B, Lawlor DA (2013). Loss to follow-up in cohort studies: bias in estimates of socioeconomic inequalities. Epidemiology.

[18] Volken T (2013). Second-stage non-response in the Swiss health survey: determinants and bias in outcomes. BMC Public Health.

[19] Lamers F, Hoogendoorn AW, Smit JH, van Dyck R, Zitman FG, Nolen WA (2012). Sociodemographic and psychiatric determinants of attrition in the Netherlands Study of Depression and Anxiety (NESDA). Compr Psychiatry.

[20] Knudsen AK, Hotopf M, Skogen JC, Overland S, Mykletun A (2010). The health status of nonparticipants in a population-based health study: the Hordaland Health Study. Am J Epidemiol.

[21] Ramsey I, de Rooij BH, Mols F, Corsini N, Horevoorts NJE, Eckert M (2019). Cancer survivors who fully participate in the PROFILES registry have better health-related quality of life than those who drop out. J Cancer Surviv.

[22] Martin J, Tilling K, Hubbard L, Stergiakouli E, Thapar A, Davey Smith G (2016). Association of Genetic Risk for Schizophrenia With Nonparticipation Over Time in a Population-Based Cohort Study. Am J Epidemiol.

[23] de Graaf R, Bijl RV, Smit F, Ravelli A, Vollebergh WAM (2000). Psychiatric and Sociodemographic Predictors of Attrition in a Longitudinal Study The Netherlands Mental Health Survey and Incidence Study (NEMESIS). Am J Epidemiol.

[24] Davis KAS, Coleman JRI, Adams M, Allen N, Breen G, Cullen B, et al. Mental health in UK Biobank – development, implementation and results from an online questionnaire completed by 157 366 participants: reanalysis. BJPsych Open. 2020;6(2):1–8. 10.1192/bjo.2019.100 PMC7176892 32026800

[25] Saunders JB, Aasland OG, Babor TF, de la Fuente JR, Grant M (1993). Development of the Alcohol Use Disorders Identification Test (AUDIT): WHO collaborative project on early detection of persons with harmful alcohol consumption–II. Addiction.

[26] Kroenke K, Spitzer RL, Williams JBW (2001). The PHQ-9. J Gen Intern Med.

[27] Spitzer RL, Kroenke K, Williams JBW, Löwe B (2006). A brief measure for assessing generalized anxiety disorder: the GAD-7. Arch Intern Med.

[28] Team RC. R: A language and environment for statistical computing. R Foundation for Statistical Computing, Vienna, Austria. 2012. 2021.

[29] Jackman S, Kleiber C, Zeileis A. Regression Models for Count Data in R. Working papers [Internet]. 2007. Faculty of Business and Economics - University of Basel.

[30] Chen SY, Feng Z, Yi X (2017). A general introduction to adjustment for multiple comparisons. J Thorac Dis.

[31] Gomez L, Díaz-Torres S, Colodro-Conde L, Garcia-Marin LM, Yap CX, Byrne EM, Yengo L, Lind PA, Wray NR, Medland SE, Hickie IB. Phenotypic and genetic factors associated with donation of DNA and consent to record linkage for prescription history in the Australian Genetics of Depression Study. Eur Arch Psychiatry Clin Neurosci. 2022;1–10. 10.1007/s00406-022-01527-0 36422680

[32] Popejoy AB, Fullerton SM (2016). Genomics is failing on diversity. Nature.

[33] Munafò MR, Tilling K, Taylor AE, Evans DM, Davey SG (2018). Collider scope: when selection bias can substantially influence observed associations. Int J Epidemiol.

[34] Larsson H. The importance of selection bias in prospective birth cohort studies. JCPP Advances. 2021;1(3):12043–5. 10.1002/jcv2.12043 PMC10242968 37431437

[35] Lemieux T, Milligan K, Schirle T, Skuterud M (2020). Initial Impacts of the COVID-19 Pandemic on the Canadian Labour Market. Can Public Policy.

[36] Pierce M, McManus S, Jessop C, John A, Hotopf M, Ford T (2020). Says who? The significance of sampling in mental health surveys during COVID-19. Lancet Psychiatry.

[37] Särndal CE, Lundström S (2010). Design for estimation: Identifying auxiliary vectors to reduce nonresponse bias. Surv Methodol.

