# Supplementary material for: Sociodemographic, mental health, and physical health factors associated with participation within re-contactable mental health cohorts: an investigation of the GLAD Study
Source: BMC Psychiatry. 2023 Jul 26;23:542. doi: 10.1186/s12888-023-04890-x (PMC10373233; doi:10.1186/s12888-023-04890-x)

Supplementary materials

## Statistical analysis

### Variable coding details

**Sociodemographic variables**

Gender: Since a low number of participants endorsed having a non-binary or prefer to self-define gender identity, these groups were collapsed together to create a three level gender variable. This includes “male”, “female” and “non-binary/prefer to self-define”.

Highest education: The following academic qualifications were measured as binary variables in the GLAD Study: university/college degree; AS/A-level; GCSEs/O-levels; CSEs; NVQ/HND/HNC. These variables were used to create a ‘highest education’ variable, where the participant was assigned to the category reflecting their highest educational qualification. If the participant did not have any of the measured academic qualifications, they were assigned to a “none of the above” category.

Partnership status: The partnership variable was reduced to three levels as the response groups were comparable. Specifically, people who reported being in a relationship and living together, being in a relationship and not living together and those who were married/in a civil partnership were grouped into one category. By contrast, participants who were divorced, separated and widowed were recorded into another category. Participants who were single were treated as their own group.

**Assessment of mental health diagnoses**

As reported in our previous analyses with the COPING and RAMP studies (Young et al., 2021), the participants’ history of prior mental health diagnoses was assessed using a checklist (Table S1). This question had the following prompt: “Have you ever been diagnosed with one or more of the following mental health problems by a professional, even if you don't have it currently? *By professional we mean: any doctor, nurse or person with specialist training (such as a psychologist, psychiatrist etc.). Please include disorders even if you did not need treatment for them or if you did not agree with the diagnosis.”*

**Table S1.** Diagnosis questionnaire response options and categorisation used in analyses

| **Individual diagnoses (response options)** | **Category** |
| --- | --- |
| Depression | Depressive disorder only |
| Depression during or after pregnancy (antenatal/postnatal depression) | Depressive disorder only |
| Premenstrual dysphoric disorder (PMDD) | Depressive disorder only |
| Anxiety, nerves or generalised anxiety disorder | Anxiety disorder only |
| Social anxiety or social phobia | Anxiety disorder only |
| Specific phobia (e.g. phobia of flying) | Anxiety disorder only |
| Agoraphobia | Anxiety disorder only |
| Panic disorder | Anxiety disorder only |
| Panic attacks | Anxiety disorder only |
| *NOTE: If both depression and anxiety were present, a separate category of comorbid depressive and anxiety disorder was created* | Depressive and anxiety disorder |
| Obsessive-compulsive disorder (OCD) | OCRDs |
| Other obsessive-compulsive related disorder e.g. skin picking | OCRDs |
| Anorexia nervosa | Eating disorders |
| Bulimia nervosa | Eating disorders |
| Psychological overeating or binge-eating disorder | Eating disorders |
| Mania, hypomania, bipolar or manic-depression | Bipolar disorder |
| Schizophrenia | Psychosis only |
| Schizoaffective disorder | Psychosis only |
| Any other type of psychosis or psychotic illness | Psychosis only |
| *NOTE: If both bipolar and psychosis were present, a separate category of comorbid psychotic and bipolar disorder was created* | Psychotic and bipolar disorder |
| Personality disorder | Personality disorder |
| Autism, asperger's or autistic spectrum disorder | ASD |
| Attention deficit or attention deficit and hyperactivity disorder (ADD/ADHD) | ADHD |

Adapted from “Depression, anxiety and PTSD symptoms before and during the COVID-19 pandemic in the UK” by Young, K. S., Purves, K. L., Hübel, C., Davies, M. R., Thompson, K. N., Bristow, S., … Breen, G, 2021, *PsyArXiv, 1*(1), supplementary materials.

**Checking outliers**

Histograms and boxplots were created to check for outliers in the continuous variables, including: age, PHQ9 (depression) sum score, GAD7 (anxiety) sum score, AUDIT (hazardous and harmful alcohol consumption) sum score, and total self-reported mental health disorders. The boxplots for age, AUDIT, and total self-reported mental health disorders suggested that some observations could be considered as outliers. However, when inspecting the histograms, it is evident that these values were ultimately a natural continuation of the distribution of the other values on these variables. Moreover, the values of these indicated cases were also plausible. Therefore, it would be inappropriate to modify or exclude these cases from the analyses.

*Age*


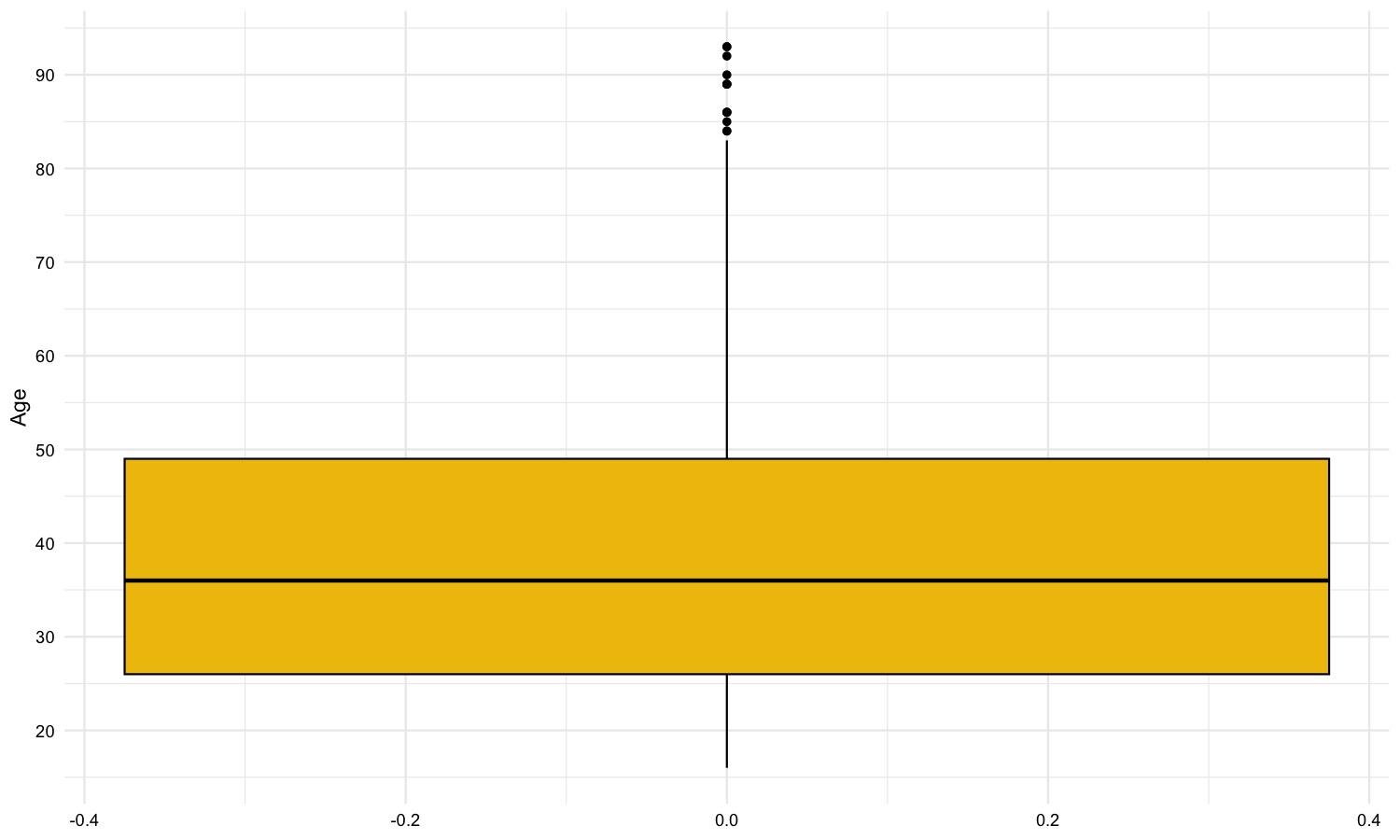


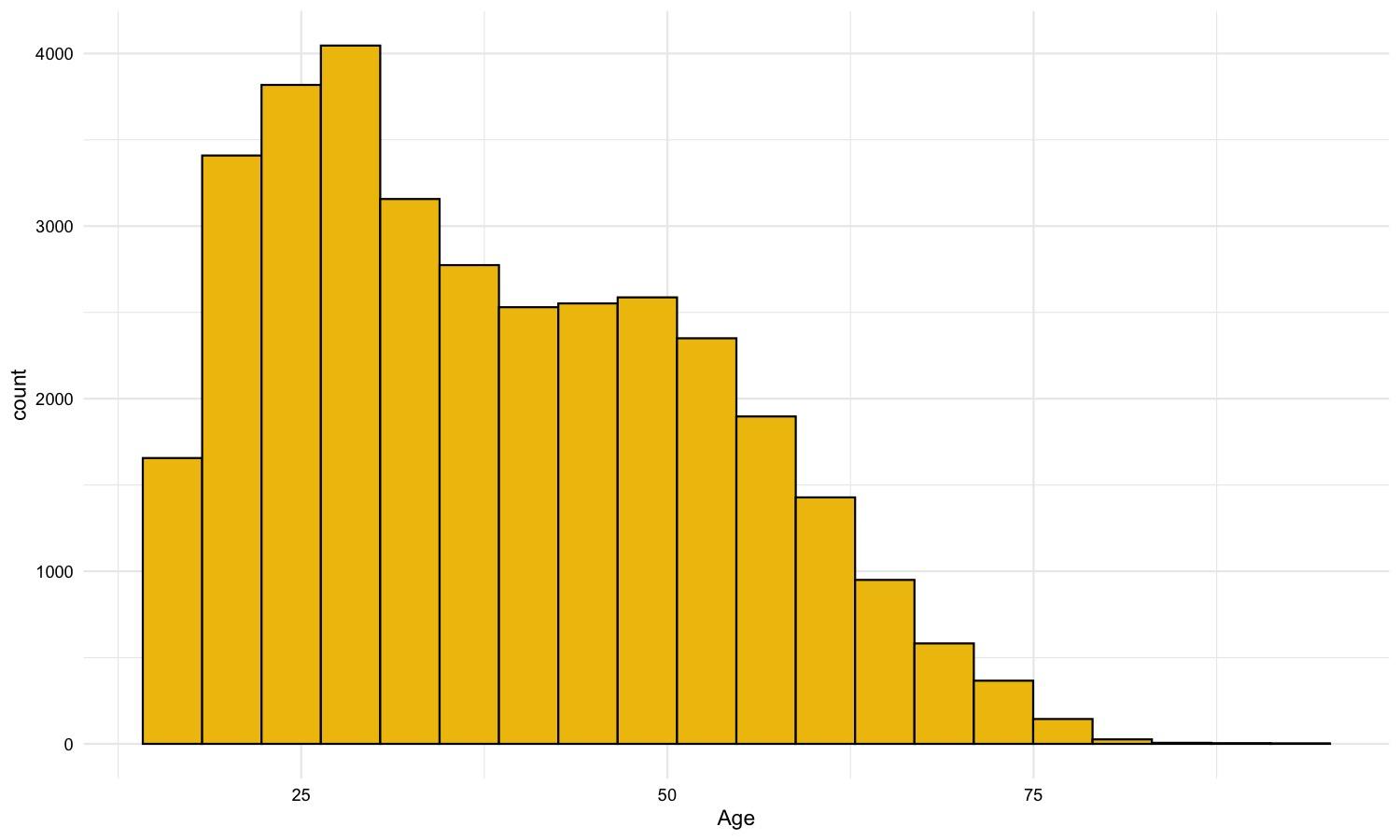


*AUDIT (alcohol consumption)*


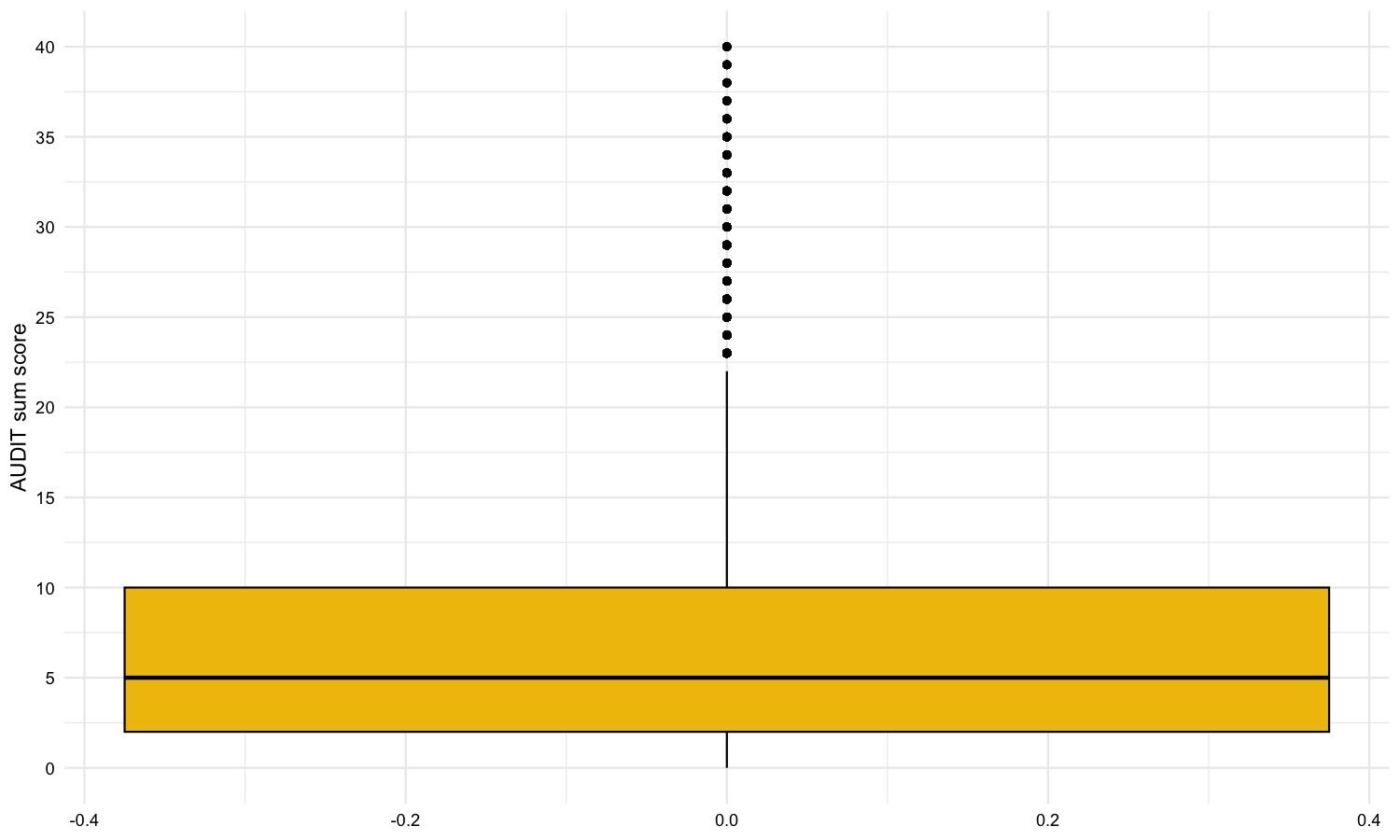


*
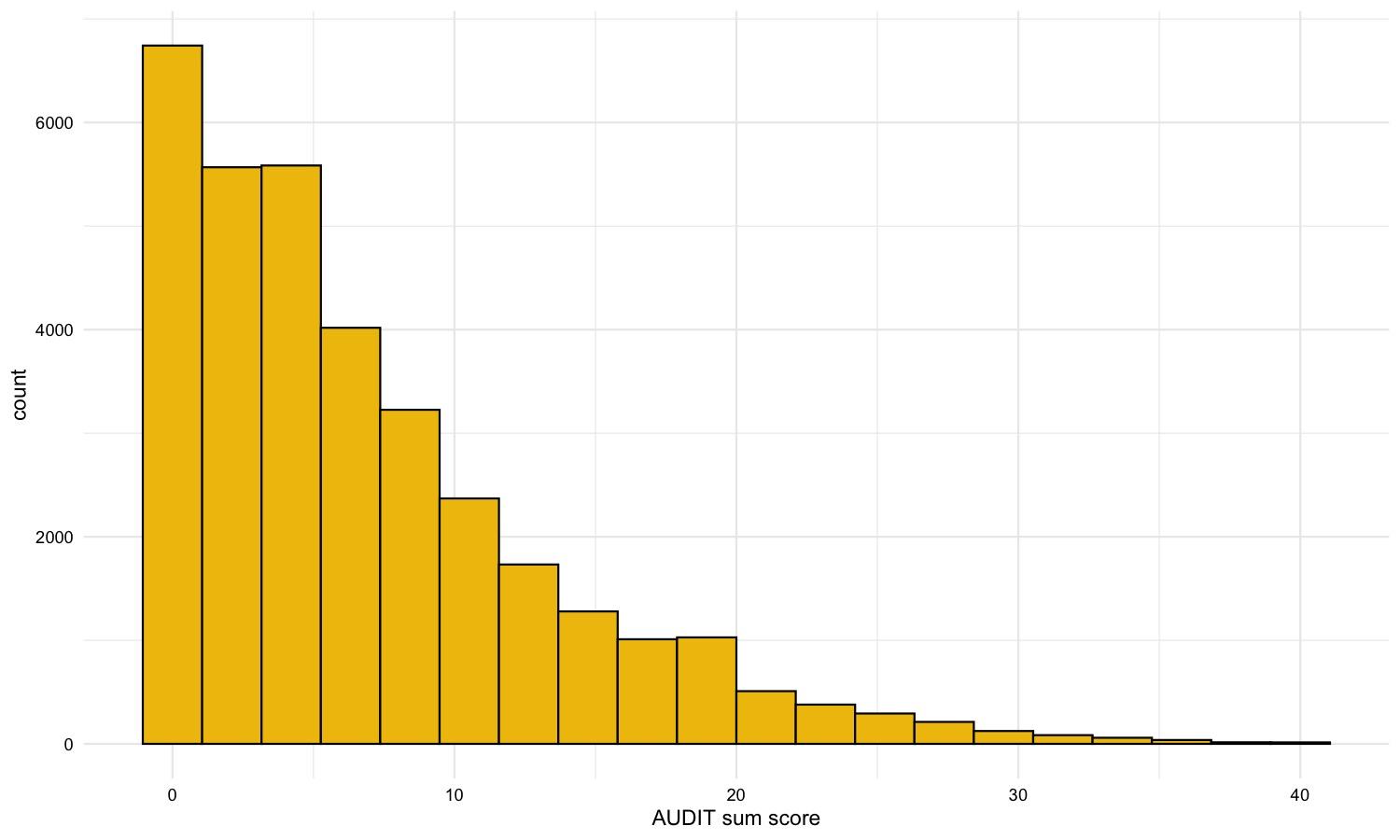
*

*GAD (anxiety) sum score*


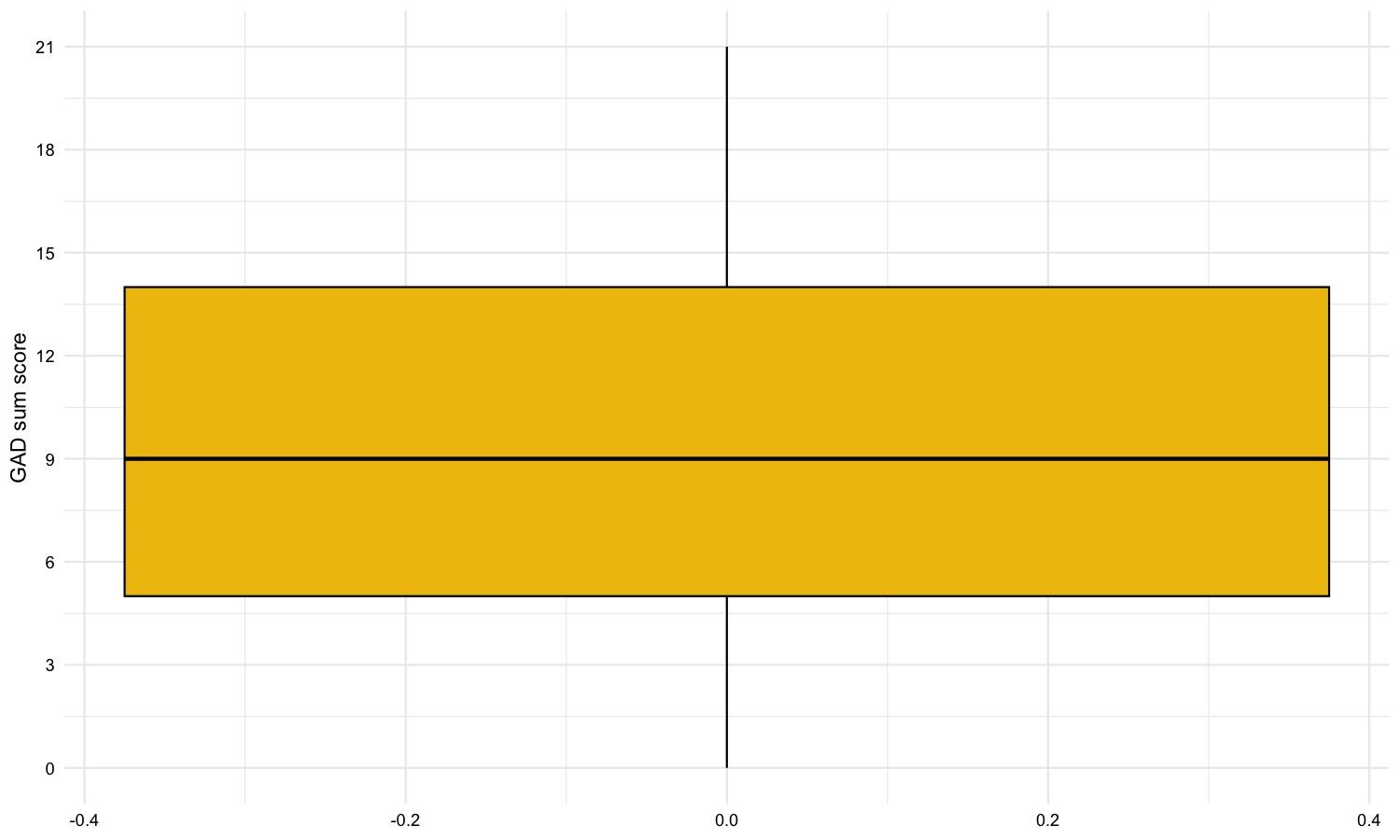


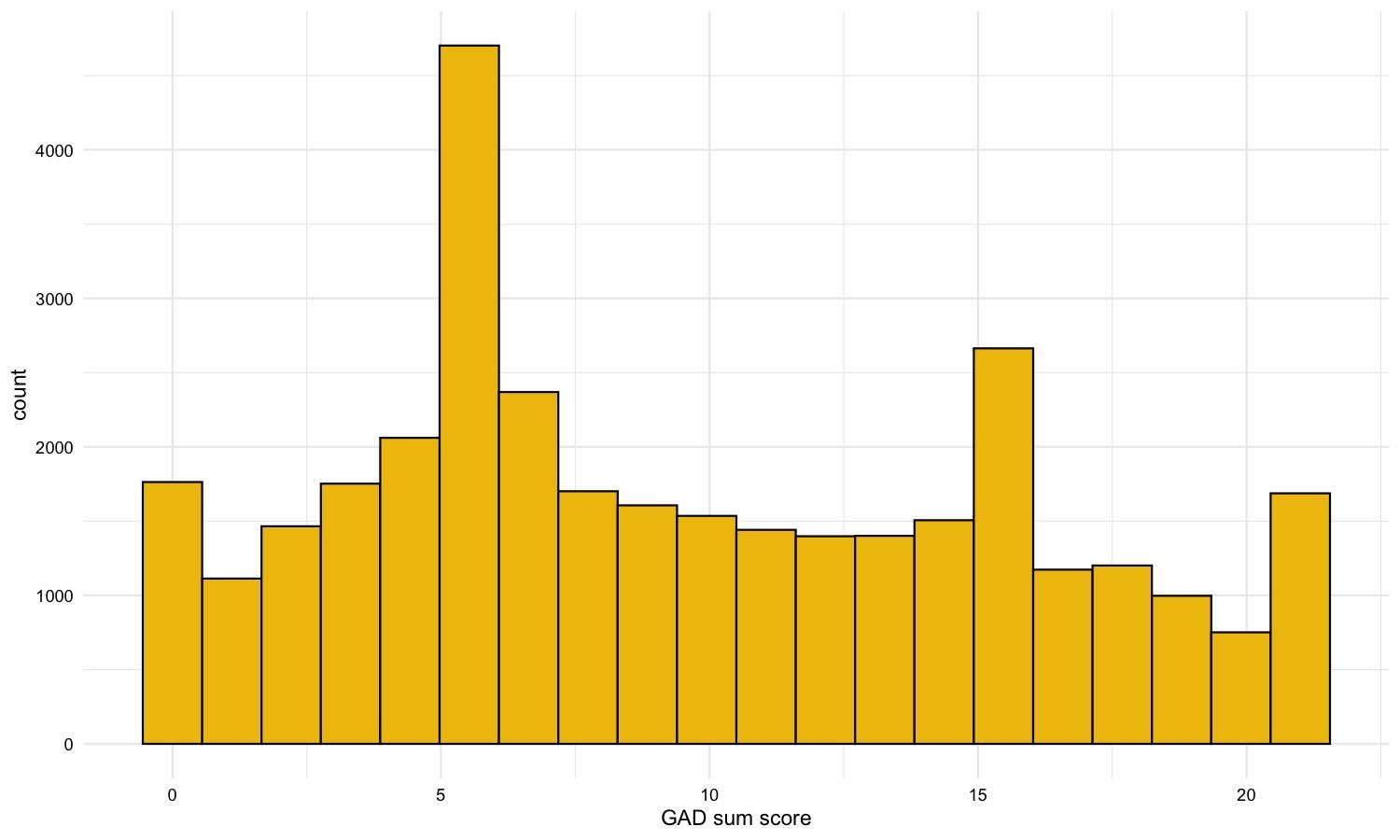


*Number of self-reported mental health disorders*


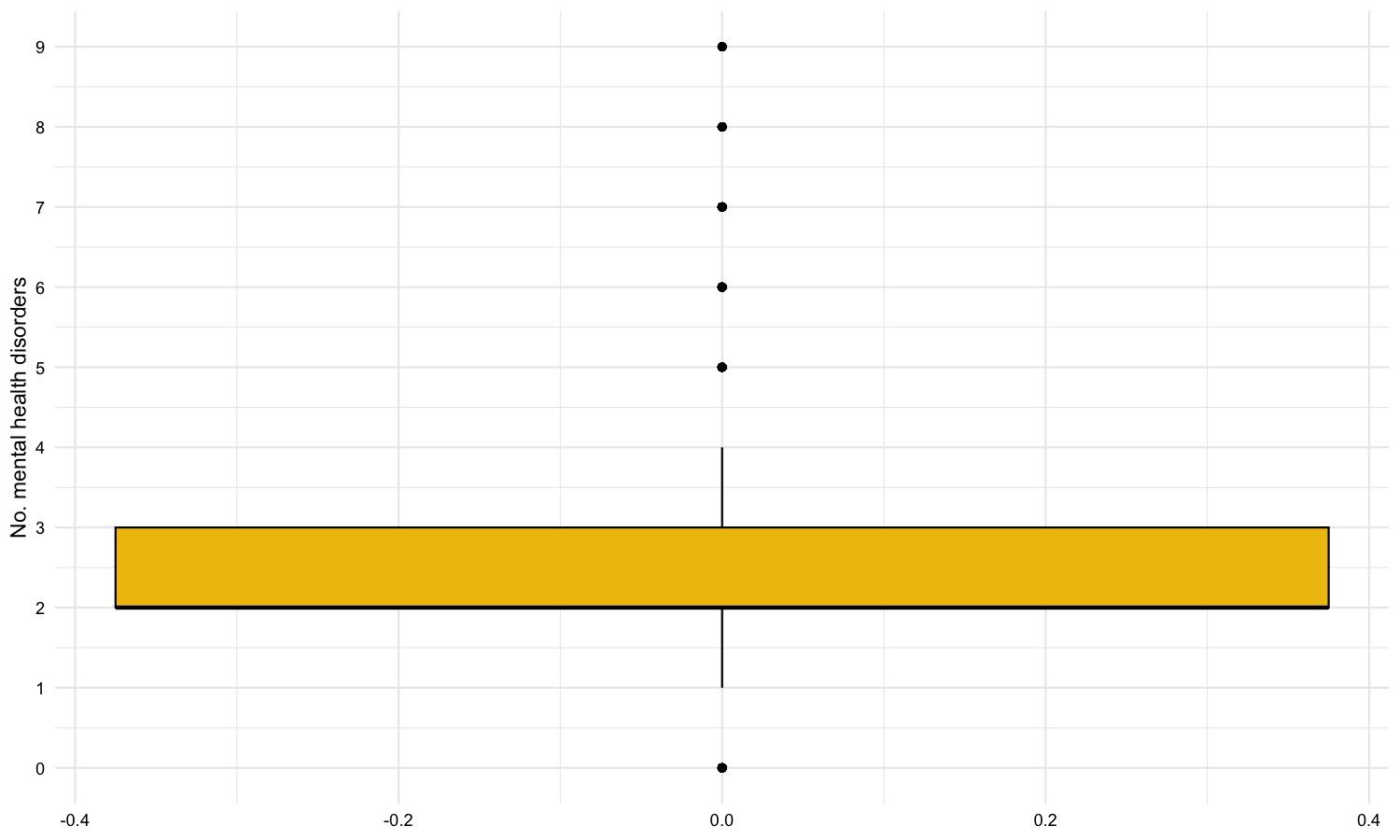


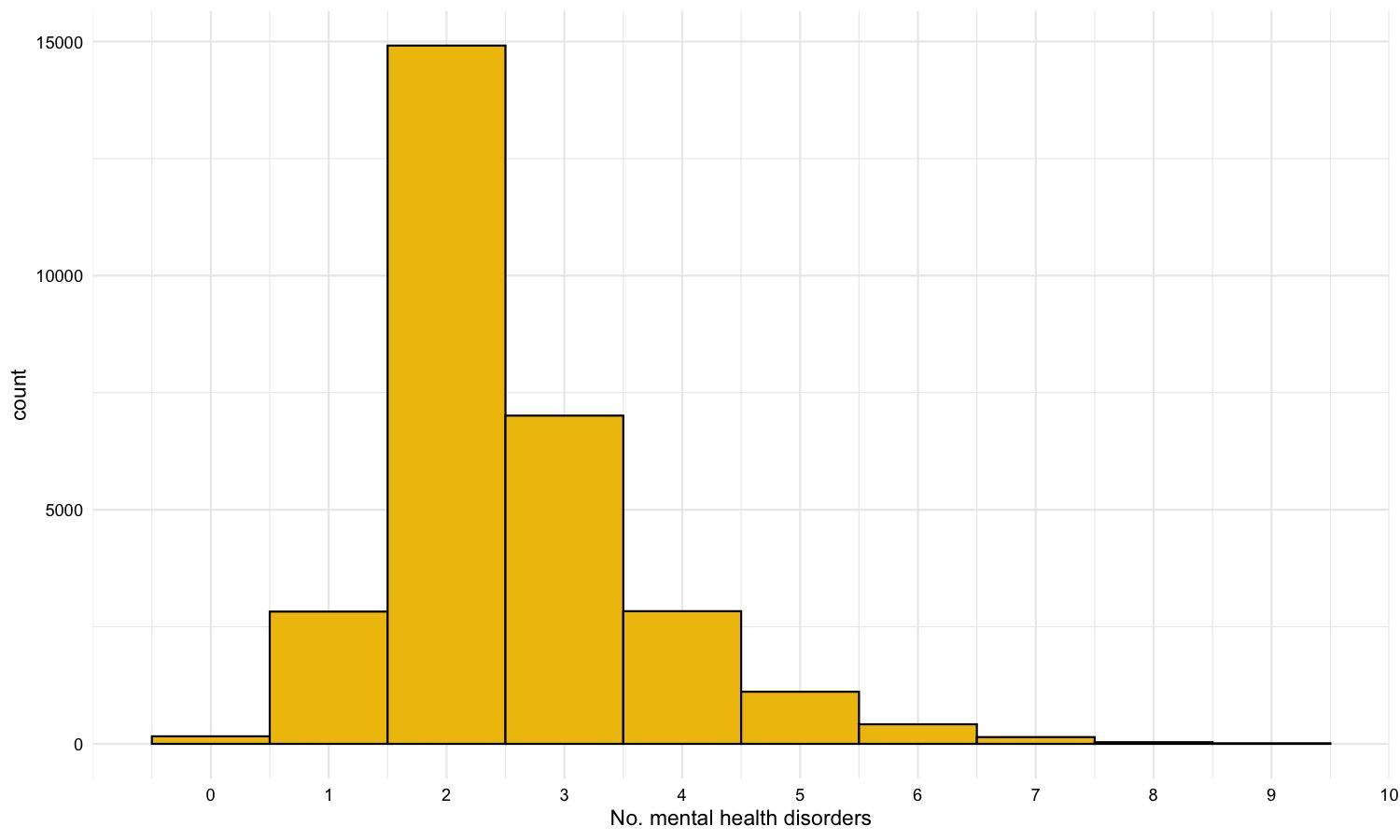


PHQ (depression) sum score


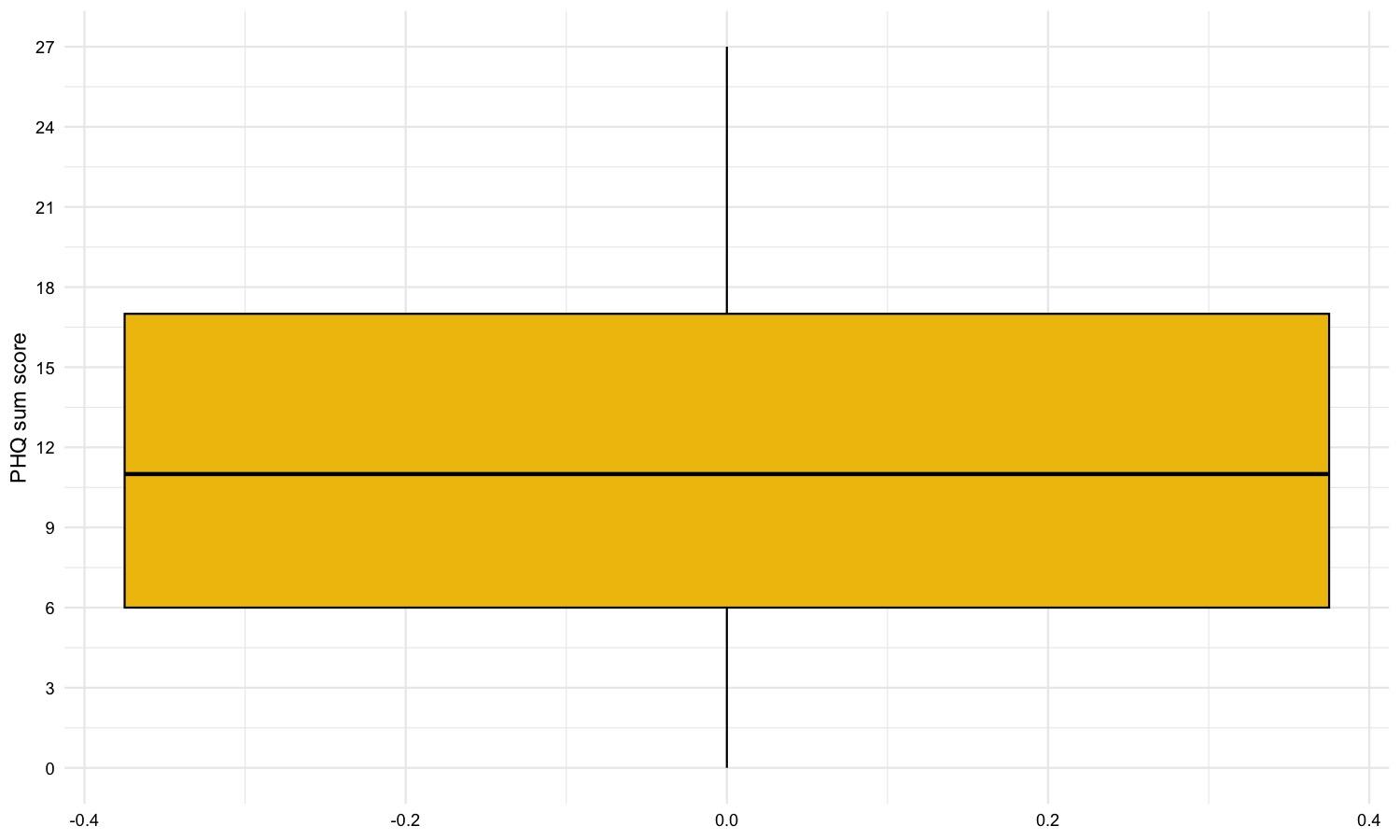


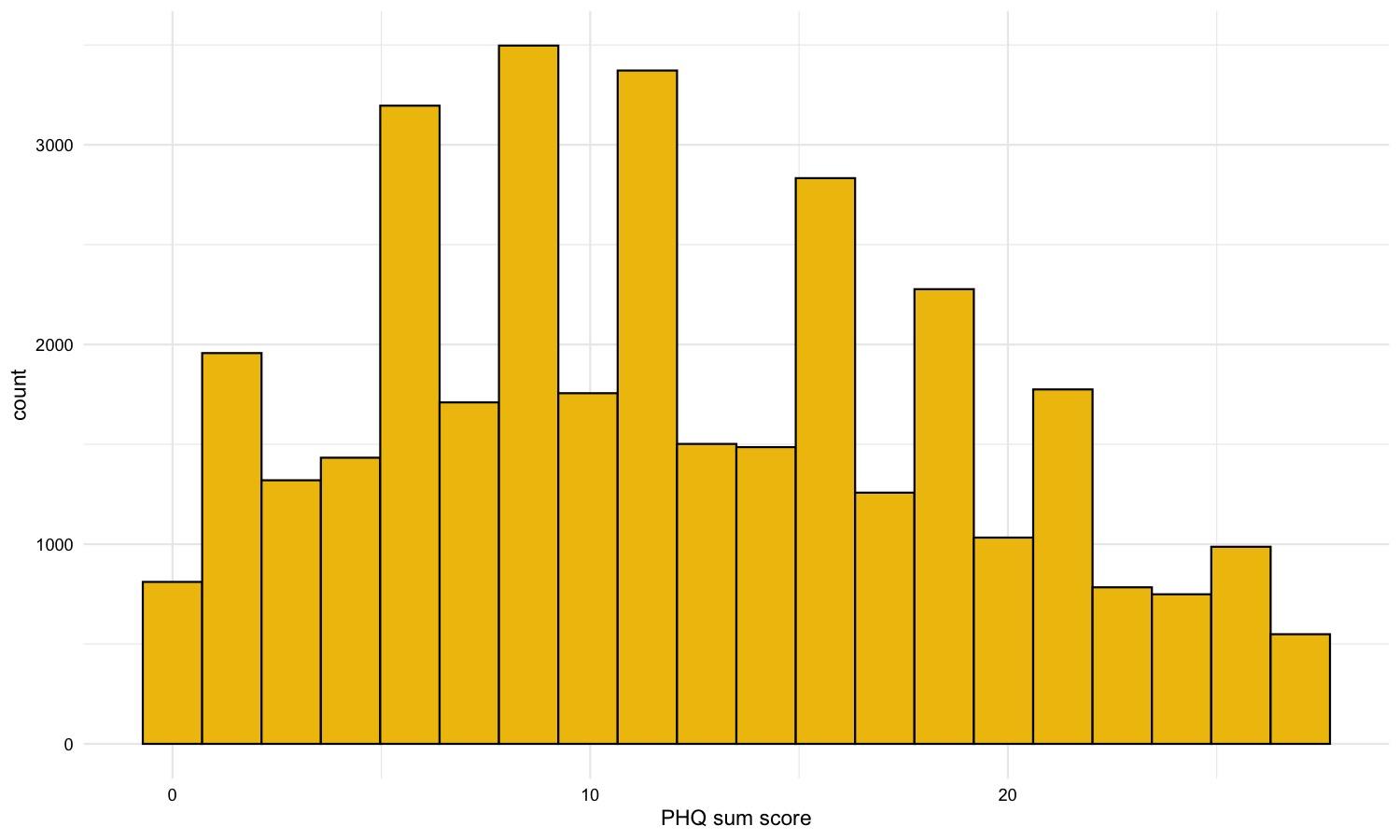


**Testing linearity assumption of logistic regression model**

Box-Tidwell tests were conducted on the continuous variables in the logistic regression model to investigate the linearity assumption (Shrestha, 2019). The results showed that age was linearly related to the logit transformation of the binary COPING baseline survey completion variable (p = 0.18). In contrast, the PHQ, GAD, AUDIT sum scores, and the total number of self-reported mental health disorders were all non-linearly related to the outcome (all p < 0.001), thus violating the assumption. Scatter plots between the log odds of COPING baseline survey completion and the continuous variables are presented below to illustrate these relationships.

To correct for this violation of the linearity assumption and assess the impact on our results, a sensitivity analysis was conducted in which the non-linearly related variables were categorised as factors, following recommendations from prior research (Long, 2008). The PHQ, GAD, and AUDIT sum score variables were categorised according to the scales' respective cut-off scoring conventions (see Kroenke et al., 2001; Saunders et al., 1993; Spitzer et al., 2006). The total mental health disorders variable was categorised to a three level factor to match the physical health disorders variable as follows: 0 mental health disorders, 1 mental health disorder, and 2 or more mental health disorders.

The results of this sensitivity analysis showed that, after Bonferonni adjustment, all the variables that were originally significant in the aim 1 logistic regression model remained significant with the same effect size direction. Notably, when AUDIT was treated as a factor, its highest level of possible dependence was significant and this was thus likely driving the association when it was treated as a continuous variable. The full model results from this sensitivity analysis can be viewed in additional file 2.

*Age*


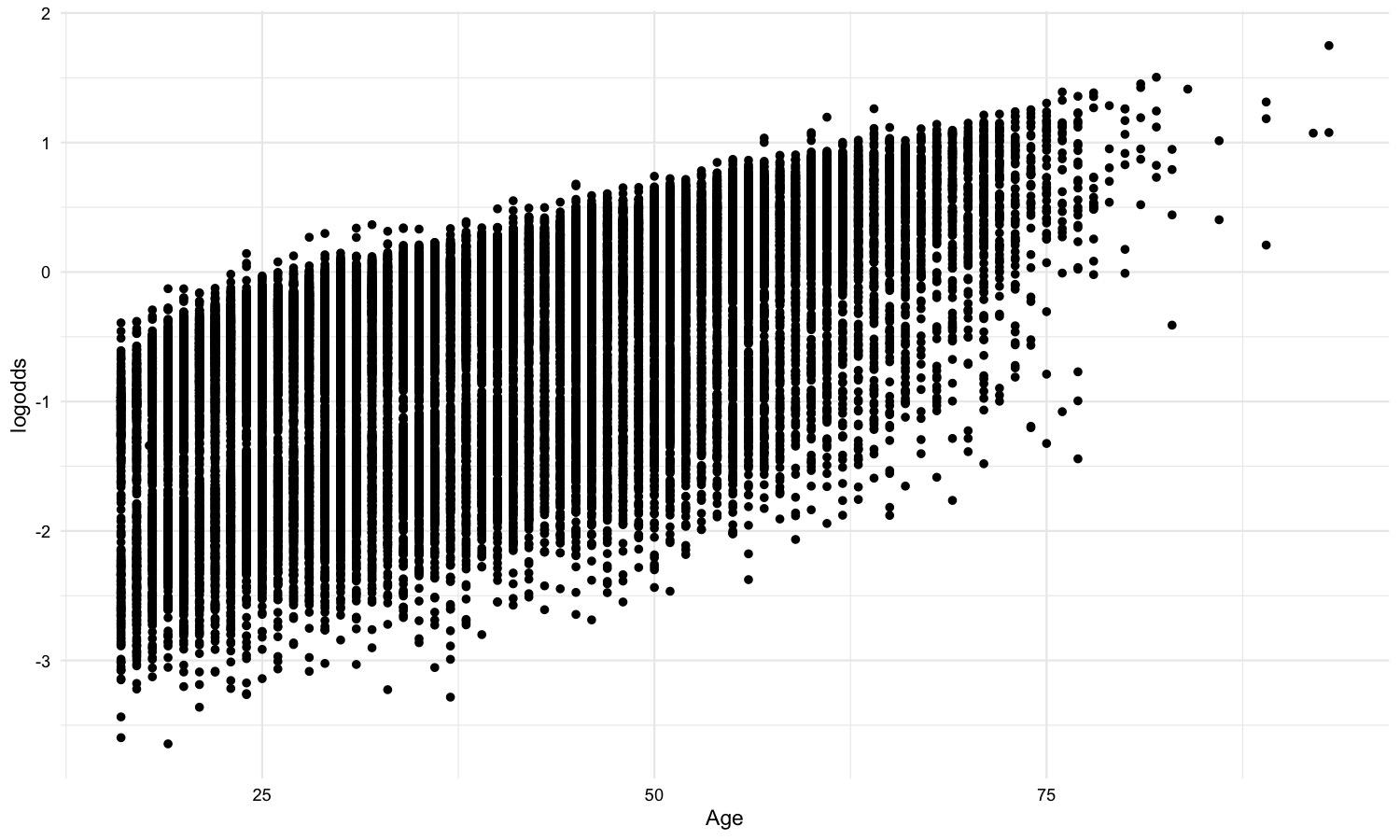


*AUDIT (alcohol consumption) sum score*


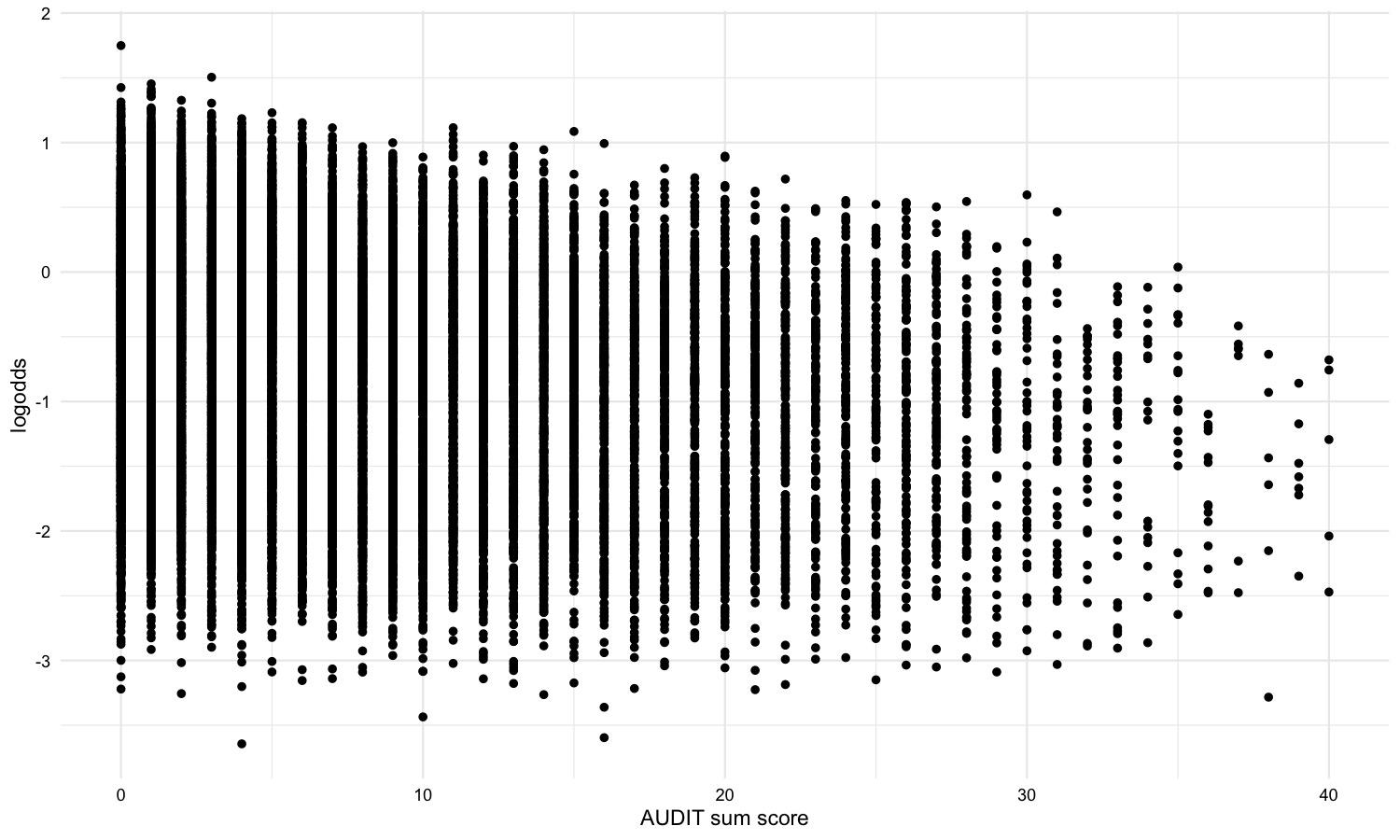


*GAD (anxiety) sum score*


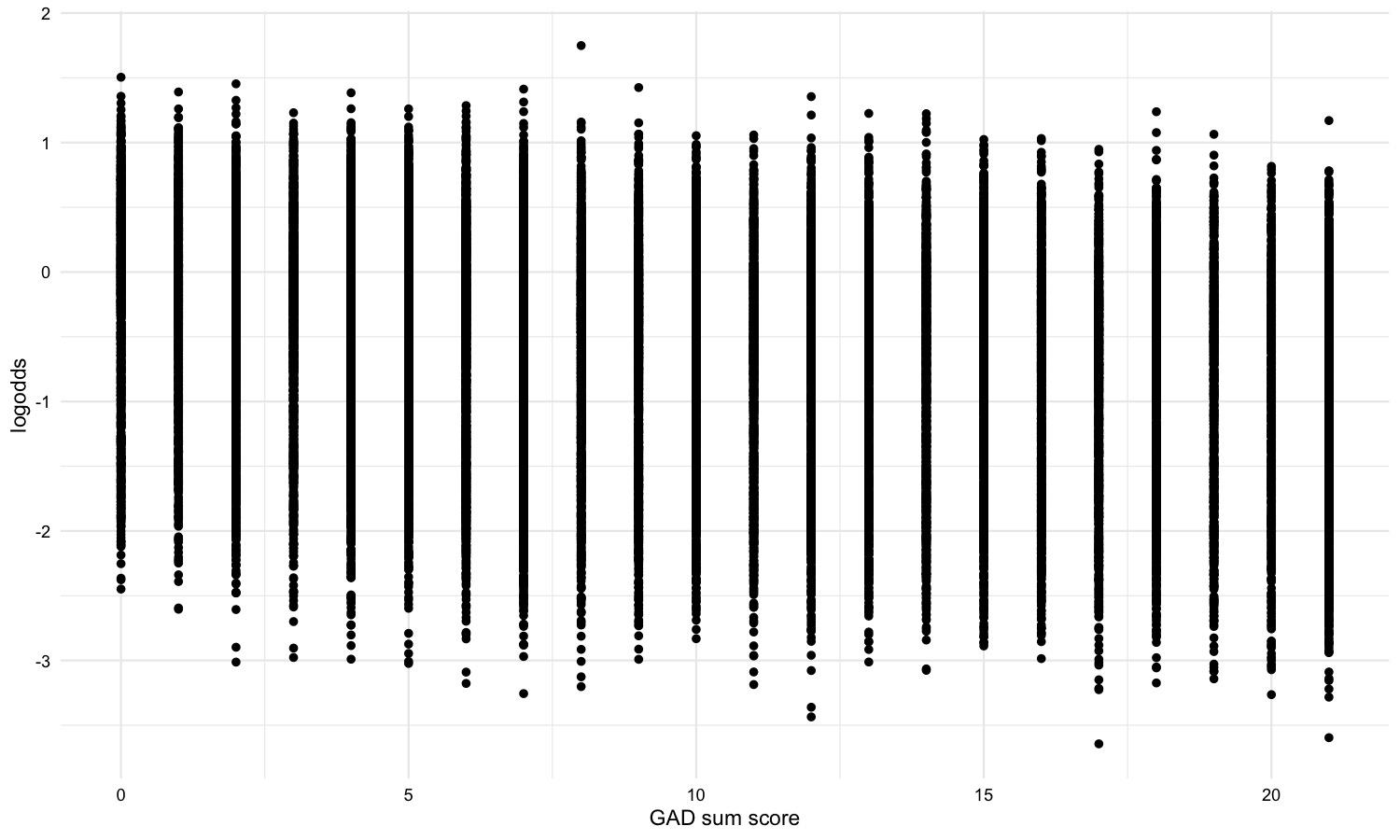


*Total mental health disorders*


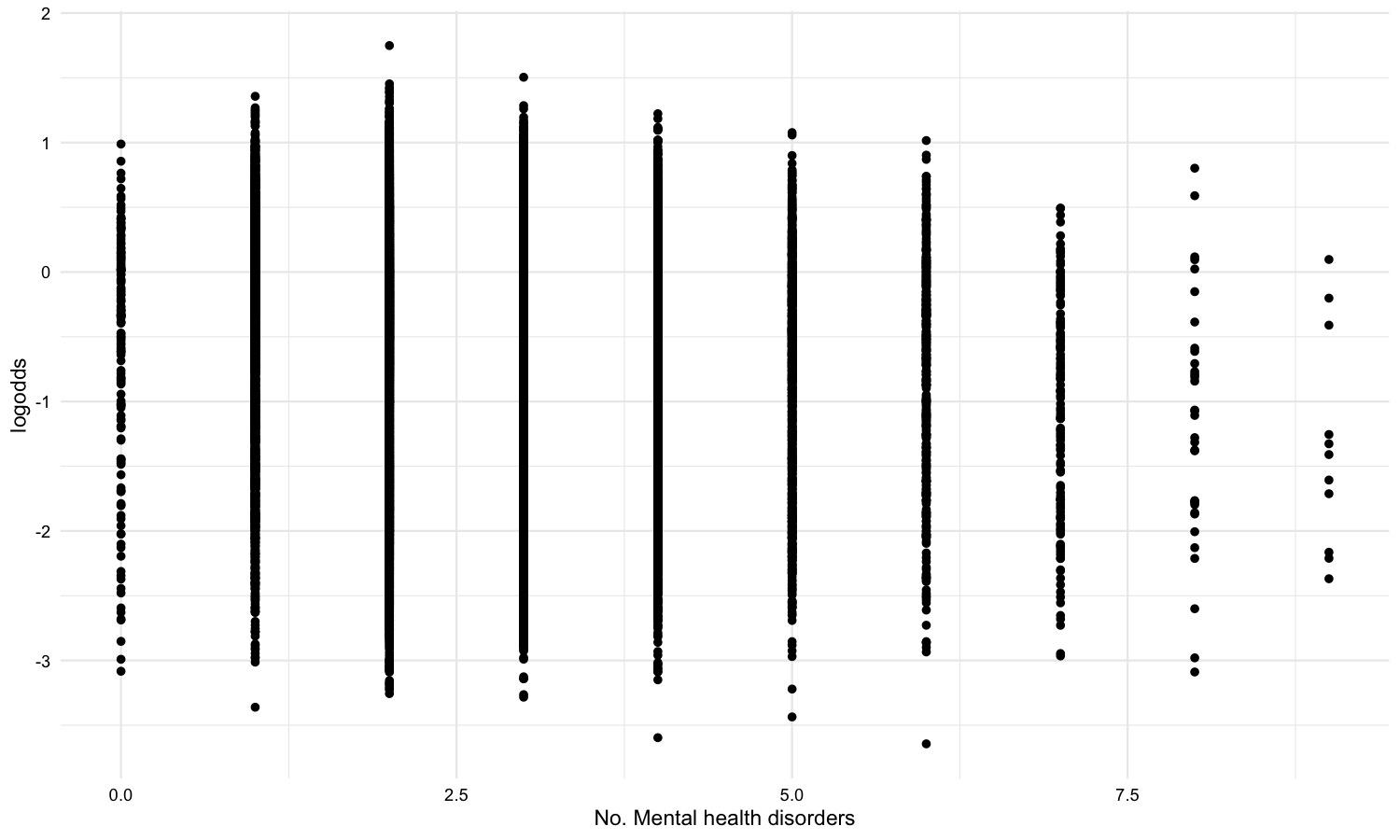


*PHQ (depression) sum score*


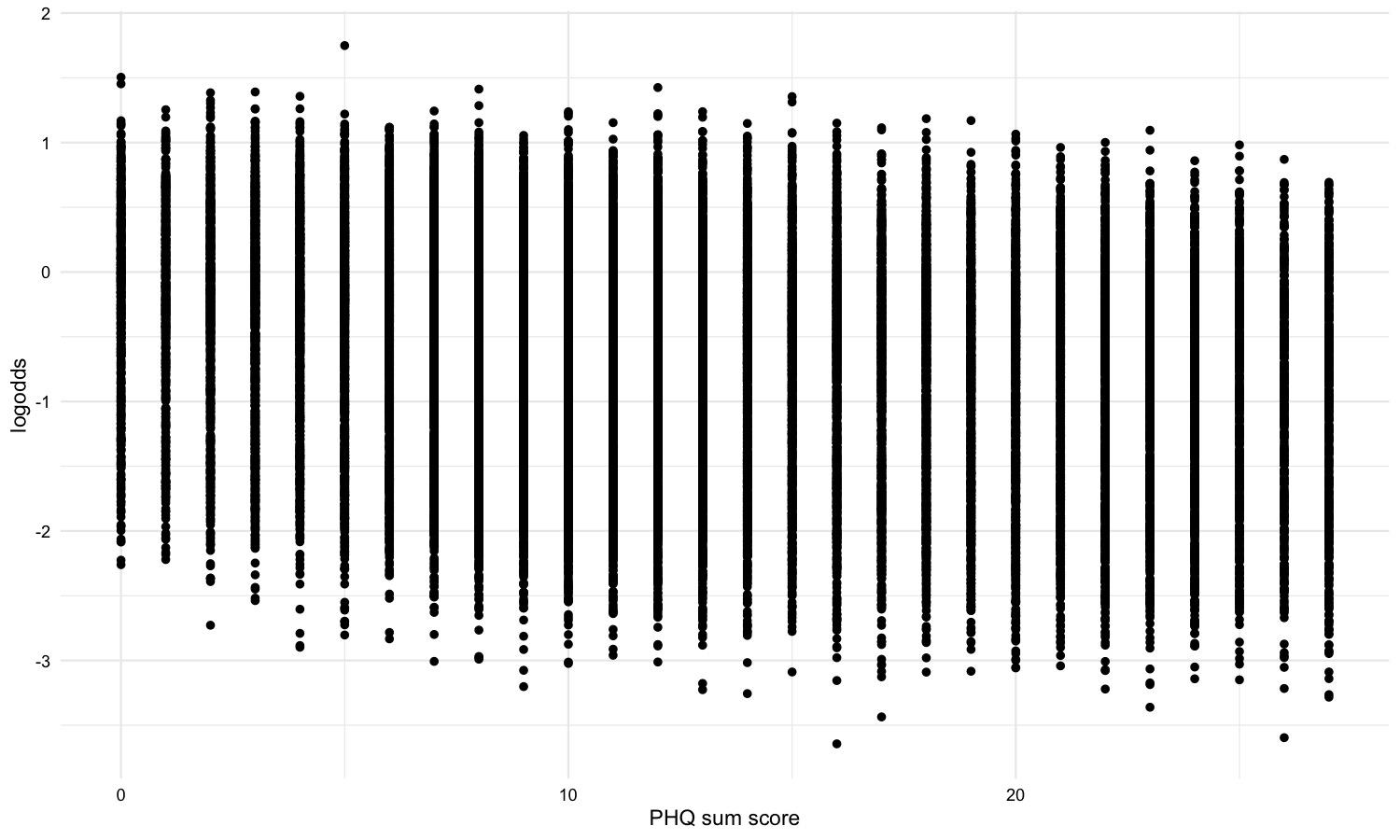


Long, R. G. (2008). The crux of the method: assumptions in ordinary least squares and logistic regression. Psychological Reports, 103(2), 431-434.

Kroenke K, Spitzer RL, Williams JBW. The PHQ-9. J Gen Intern Med. 2001 Sep;16(9):606–13.

Saunders JB, Aasland OG, Babor TF, de la Fuente JR, Grant M. Development of the Alcohol Use Disorders Identification Test (AUDIT): WHO collaborative project on early detection of persons with harmful alcohol consumption--II. Addiction. 1993 Jun;88(6):791–804.

Shrestha, N. (2019). Application of binary logistic regression model to assess the likelihood of overweight. Am J Theor Appl Stat, 8(1), 18-25.

Spitzer RL, Kroenke K, Williams JBW, Löwe B. A brief measure for assessing generalized anxiety disorder: the GAD-7. Arch Intern Med. 2006 May 22;166(10):1092–7.

##

## Results

Correlations between the sociodemographic, mental health, physical health, and participation variables.

The correlation between completing the COPING baseline survey and the number of completed COPING follow-up surveys is null because the standard deviation is zero. That is, only those who take part in the COPING baseline survey would have completed follow-up surveys.


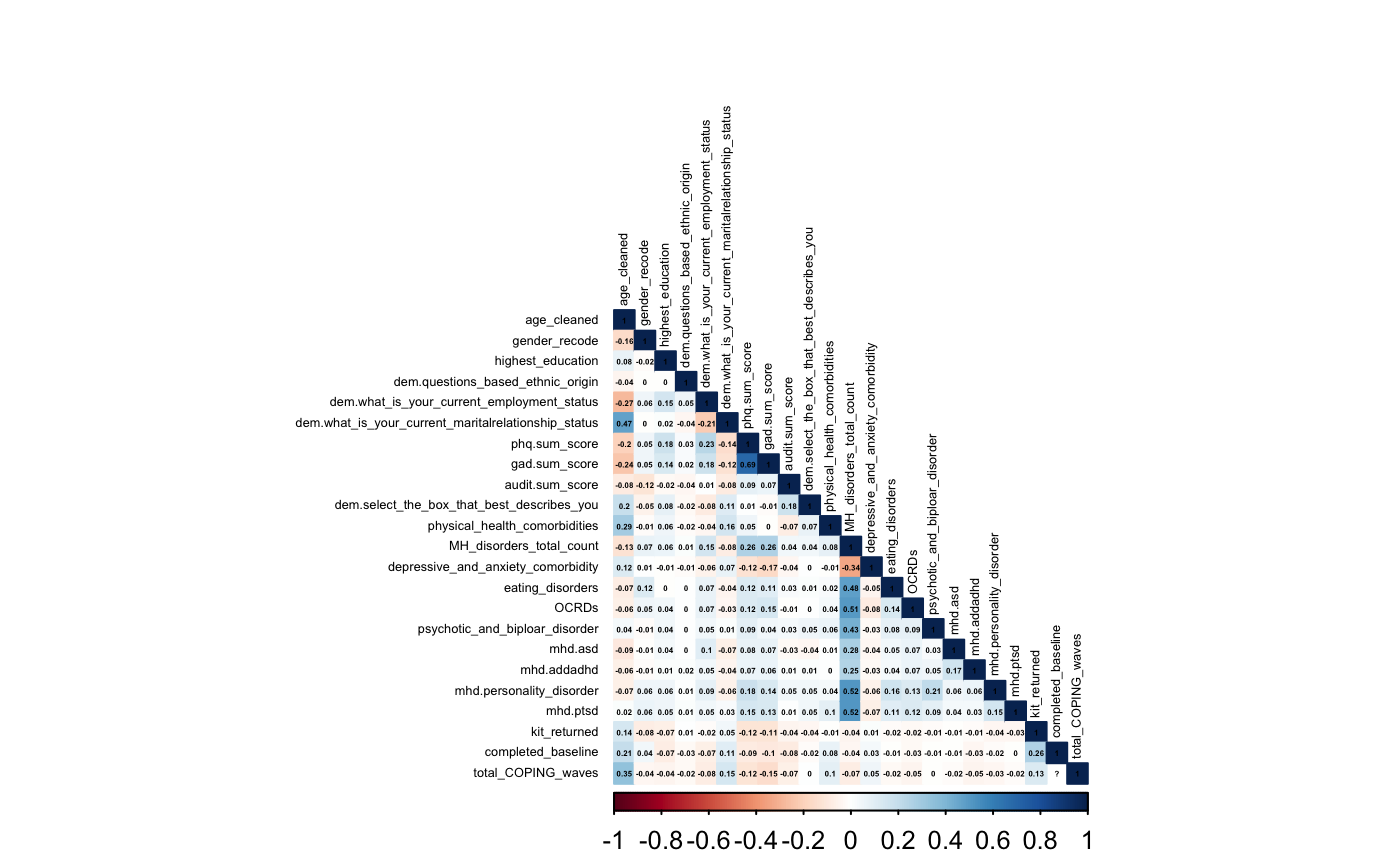

Supplement: Supplementary file 1 — Additional file 1: Further methodological information and variable correlation results. [file 12888_2023_4890_MOESM1_ESM.docx]
